# Supplementary material for: South Asian black carbon is threatening the water sustainability of the Asian Water Tower
Source: Nat Commun. 2022 Nov 30;13:7360. doi: 10.1038/s41467-022-35128-1 (PMC9712424; doi:10.1038/s41467-022-35128-1)
Supplement: Supplementary file 1 — Supplementary materials [file 41467_2022_35128_MOESM1_ESM.pdf]

Supplementary Information for:

South Asian black carbon is threatening the water sustainability of the Asian Water Tower

Junhua Yang[1], Shichang Kang[1, 2]*, Deliang Chen[3], Zhao Lin[4], Zhenming Ji[5], Keqin Duan[6], Haijun Deng[7], Lekhendra Tripathee[1], Wentao Du[1], Mukesh Rai [1], Fangping Yan[1], Yuan Li[1], Robert R. Gillies[8, 9]*

[1]State Key Laboratory of Cryospheric Sciences, Northwest Institute of Eco-Environment and Resources, Chinese Academy of Sciences, Lanzhou,730000, China

[2]University of Chinese Academy of Sciences, Beijing 100049, China

[3]Regional Climate Group, Department of Earth Sciences, University of Gothenburg, Gothenburg, 40530, Sweden

[4]Key Laboratory of Land Surface Process and Climate Change in Cold and Arid Regions,Chinese Academy of Sciences, Lanzhou,730000, China

[5]School of Atmospheric Sciences, Key Laboratory of Tropical Atmosphere - Ocean System, Ministry of Education, Sun Yat-sen University, Zhuhai, 519082, China

[6]School of Geography and Tourism, Shaanxi Normal University, Xi'an 710119, China

[7]School of Geographical Sciences, Fujian Normal University, Fuzhou 350007, China

[8]Utah Climate Center, Utah State University, Logan, UT, USA

[9]Department of Plants, Soils, and Climate, Utah State University, Logan, UT, USA

Correspondence：Shichang Kang shichang.kang@lzb.ac.cn or Robert R. Gillies: robert.gillies@usu.edu

Contents

Introduction

1. Methods

1.1 The accumulative anomaly method

1.2 Model evaluation statistics

1.3 WRF-Chem setup and evaluation

1.3.1 WRF-Chem setup

1.3.2 Evaluation of precipitation

1.3.3 Evaluation of black carbon

1.3.4 Model uncertainty analysis

1.4 Glacier mass balance model

2. Precipitation and incoming moisture variations over the Tibetan Plateau

2.1 Precipitation trends over the Tibetan Plateau

2.2 Change of incoming moisture from South Asia to the southern Tibetan Plateau

3. South Asian black carbon emissions, loading, and atmospheric heating

4. South Asian black carbon linked with summer precipitation decrease in south Tibetan Plateau

4.1 The lag correlation analysis between black carbon and summer precipitation

4.2 Mechanism of South Asian black carbon affecting on precipitation

5. Glacial mass balance change and its climate drivers

5.1 Glacial mass balance change over the Tibetan Plateau

5.2 Drivers of glacial mass balance change

6. Supplementary information for datasets used in this work

7. Supplementary References

Introduction

We describe the methods in more details in Section 1, including the accumulative anomaly methodology, model evaluation statistics, WRF-Chem model set up with an in-depth simulation evaluation and uncertainty analysis, as well as a monthly-scale glacial mass balance model. Next, we discuss precipitation and incoming moisture variations over the Tibetan Plateau, including precipitation trends over the Tibetan Plateau and changes in incoming moisture from South Asia to the southern Tibetan Plateau (Section 2). South Asian black carbon emissions, loadings, and atmospheric heating, are described in Section 3. To better explain mechanistically the relationship between black carbon and the change in the water vapor transport to the Tibetan Plateau, we have added a calculation of moisture flux and its divergence from 1989 to 201 (Section 4). Lastly, in Section 5, we have added additional analyses to support the key conclusions with respect to glacial retreat over the Tibetan Plateau; this includes (i) a reconstruction time series of reference-surface mass balance from 1979 to 2014 using an empirical model, (ii) calculated contributions of summer precipitation and temperature change to glacier mass change, as well as (iii) its spatial heterogeneity. An itemized summary of the datasets used in this study is provided in Section 6.

1. Methods

1.1 The accumulative anomaly method

The accumulative anomaly method was used to reveal the inflexion point (year) where the change in precipitation occurred "due to" black carbon emission accumulation. The accumulative anomaly is an index to distinguish the changing tendency of discrete data[1]. For a discrete series, the accumulative anomaly (X_t) for data point can be expressed as:

$$X_t = \sum_{i=1}^{t}(x_i - \bar{x}) \quad t = 1, 2, \ldots, n \tag{1}$$

where $\bar{x}$ is the mean value of the series x_i, and n is the number of discrete points. A positive accumulative anomaly indicates that the corresponding data point is higher than the average, otherwise lower than the average. If the relation curve is composed of least two parts as noted, then the inflexion point/year can be identified. Here, the variable x represents summer precipitation, or black carbon emission.

1.2 Model evaluation statistics

We examined the skill of model performance utilizing in-situ observations,

satellite data, and a reanalysis dataset vs. model output. Point-based propagation was assessed for precipitation and black carbon. The following statistical metrics[2,3] were used in the study to evaluate the suitability of different physics and chemical schemes for a wide range of scenarios and applications.

The bias was calculated as the difference between the WRF-Chem simulations, the observed precipitation, and black carbon. The mean error (*ME*, equation (2)) was derived using all sites in the study area.

$$ME = \frac{1}{n}\sum_{i=1}^{n}(x_i - y_i) \tag{2}$$

Mean absolute error (*MAE*, equation (3)) was calculated to quantify the difference between the simulations and the observational data while negating the effect of cancelling positive and negative errors seen in the bias.

$$MAE = \frac{1}{n}\sum_{i=1}^{n}|x_i - y_i| \tag{3}$$

Root mean square error (*RMSE*, equation (4)), which is a measure of the combination of systematic and random error, was calculated from the simulated results.

$$\text{RMSE} = \sqrt{\frac{1}{n}\sum_{i=1}^{n}(y_i - x_i)^2} \tag{4}$$

Pearson's correlation coefficient (*R*, equation (5)) was calculated to identify the linear dependence between the simulations and the observations with a value between –1 and 1. A value of 1 implies that there is a perfect equation to describe the relationship between the simulations and observations, i.e., all data points lie on a line. The value of –1 implies a decreasing linear relationship, i.e., the simulated values decrease with increasing observed values. The values between the two imply the degree of linear relationship between the simulations and observations.

$$R = \frac{\frac{1}{n}\sum_{i=1}^{n}(x_i-\bar{x})(y_i-\bar{y})}{\sqrt{\sum_{i=1}^{n}(x_i-\bar{x})^2(y_i-\bar{y})^2}} \tag{5}$$

Slope/linear regression (equation (6)) goes one step beyond the correlation coefficient in identifying the linear relationship between the simulations and observations. This statistic metric is utilized to further calculate the total model performance (*TMP*).

$$slope = \frac{\sum_{i=1}^{n}(x_i-\bar{x})(y_i-\bar{y})}{(x_i-\bar{x})^2} \tag{6}$$

TMP (equation (7)) is a combined index derived from *MAE*, *RESM*, and the *slope* to

quantify the overall performance so that each simulation ensemble member could be compared individually.

$$TMP = \left(\frac{MAE+RMSE}{\bar{x}} + (1 + \mathrm{R})\right) + \frac{|1+slope|}{4} \tag{7}$$

Here, the metric was calculated, where n, y_i and x_i are the number of observations, simulation value, and observation value, respectively. $\overline{x}$ is the mean of observed values and $\overline{y}$ is the mean from the simulations.

1.3 WRF-Chem setup and evaluation

1.3.1 WRF-Chem setup

The WRF-Chem model is a newly developed regional dynamic/chemical transport model[4] which simulates gas-phase chemical and aerosol microphysical processes, along with numerous meteorological fields[5]. In this study, the WRF-Chem simulations were conducted at 25 km horizontal resolution, covering the study domain, with 190 grid cells in the west-east direction and 160 in the north-south direction. The detailed model setup is listed in Supplementary Table 1. The initial and boundary conditions for the meteorological fields were obtained from the 6-h National Centers for Environmental Prediction final analysis data (NCEP FNL) with a horizontal resolution of 1° × 1°. The default (initial) use of anthropogenic emissions were obtained from the Intercontinental Chemical Transport Experiment Phase B (INTEX-B); these included CO, SO_2, NO_X, VOC, BC, OC, $PM_{2.5}$ and PM_{10} and, were subsequently replaced by the real-time output of Model for OZone and Related chemical Tracers (MOZART)[6] at 6-h resolution. Successive initial chemical conditions were updated by the real-time MOZART results. Biogenic emissions were centered upon the Model of Emission of Gases and Aerosol from Nature (MEGAN)[7]. Fire emissions were obtained from the Fire INventory from NCAR (FINN)[8]. Details on the emission datasets used in the WRF-Chem simulations are summarized in Supplementary Table 2 here.

Supplementary Table 1 Detailed setup information of WRF-Chem simulations.

Map and grids	
Map projection	Lambert
Central point of study domain	26°N, 80°E
Vertical layers	35 eta levels up to 50 hPa
Horizontal grid spacing	25 Km
Initial and boundary meteorological conditions	6-hour National Centers for Environmental Prediction (NCEP) FNL analysis data
Updated initial and boundary condition	MOZART-4/GEOS-5
**Physics Options**	
Microphysics	Lin et al. scheme
Cumulus parameterization	Kain-Fritsch scheme
Shortwave radiation	RRTMG scheme
Longwave radiation	RRTMG scheme
Land surface	Unified Noah land surface model
Planetary boundary layer	YSU scheme
**Chemical scheme Options**	
Gas-phase chemistry	CBMZ scheme
Aerosol reactions scheme	MOSAIC scheme

Supplementary Table 2 Summary of different emission datasets used in WRF-Chem simulations as well as their changed in different experiment.

Emission datasets	Resolutions		Changes in experiments	
	Spatial	Temporal	Control	Sensitivity
MEGAN biogenic emissions	0.03°	Month	Original values	Set to zero over South Asia
FINN fire emissions	500m	Hour	Original values	Set to zero over South Asia
MOZART used for anthropogenic emissions	1°	6hour	Original values	Set to zero over South Asia

There are the two generic combination optional chemical schemes for the WRF-Chem model, i.e., the CBMZ (Carbon Bond Mechanism version Z)[9] gas-phase chemical mechanism and, the MOSAIC (Model for Simulating Aerosol Interactions and Chemistry)[10] aerosol module. Additional modules include the RADM2 (Regional Acid Deposition Model v2)[11] gas-phase chemical mechanism and the MADE/SORGAM (Modal Aerosol Dynamics Model for Europe/Secondary Organic Aerosol Model)[12] aerosol scheme. In a previous study, we found the CBMZ gas-phase chemical mechanism and the MOSAIC aerosol module were more suitable for the reconstruction of aerosols concentrations over the Tibetan Plateau and adjacent regions[13]. The CBMZ gas-phase mechanism contains 67 species and 164 reactions in a lumped structure approach that classifies organic compounds according to their internal bond types[14]. Rates for photolytic reactions are modified as described in DeMore et al[15]. The MOSAIC aerosol module includes sulfate, nitrate, ammonium, sodium, calcium, chloride, black carbon, primary organic mass, liquid water, and other inorganic mass with 4 Bin size ranges: (1) 0.04–0.156 μm; (2) 0.156–0.625 μm; (3) 0.625–2.5 μm; and (4) 2.5–10.0 μm. This mechanism simulates major aerosol processes such as thermodynamic equilibrium, condensation, binary nucleation, and coagulation.

The selection of physical schemes was based on the performance of 12 WRF-Chem model configurations, created using different physical scheme combinations. As listed in Supplementary Table 3, three different treatments of cumulus parameterizations (Kain-Fritsch, Betts-Miller-Janjic, and Grell-Devenyi (GD) Ensemble Schemes), two different microphysical options (Morrison 2-moment and Lin et al. schemes), and two different planetary boundary layer options (Yonsei University (YSU) and Mellor-Yamada-Janjic (MYJ) Schemes) were evaluated. Although none of the configurations were 100% in agreement with the observations, a combination of Lin et al. microphysical scheme[16], Kain-Fritsch cumulus scheme[17], YSU planetary boundary layer scheme[18], as well as CBMZ gas-phase and MOSAIC aerosol options had the highest simulation accuracy. Therefore, we selected the model configuration #3 (Supplementary Table 1). We present the evaluation of the performance of these 12 WRF-Chem model configurations in Sections 1.3.2 and Section 1.3.3.

Supplementary Table 3 Model configuration used in the physical and chemical schemes.

Ensemble ID	Cumulus scheme	Microphysical scheme	Boundary layer scheme	Chemical scheme
1	Kain-Fritsch	Lin et al.	MYJ	CBMZ and MOSAIC
2	Kain-Fritsch	Morrison 2-moment	MYJ	CBMZ and MOSAIC
3	Kain-Fritsch	Lin et al.	YSU	CBMZ and MOSAIC
4	Kain-Fritsch	Morrison 2-moment	YSU	CBMZ and MOSAIC
5	Betts-Miller-Janjic	Lin et al.	MYJ	CBMZ and MOSAIC
6	Betts-Miller-Janjic	Morrison 2-moment	MYJ	CBMZ and MOSAIC
7	Betts-Miller-Janjic	Lin et al.	YSU	CBMZ and MOSAIC
8	Betts-Miller-Janjic	Morrison 2-moment	YSU	CBMZ and MOSAIC
9	GD Ensemble	Lin et al.	MYJ	CBMZ and MOSAIC
10	GD Ensemble	Morrison 2-moment	MYJ	CBMZ and MOSAIC
11	GD Ensemble	Lin et al.	YSU	CBMZ and MOSAIC
12	GD Ensemble	Morrison 2-moment	YSU	CBMZ and MOSAIC

1.3.2 Evaluation of precipitation

To assess the ability of the WRF-Chem model to reproduce the summer precipitation spatial distribution, we compared simulated output with other gridded datasets including GPCP (Global Precipitation Climatology Project)[19], CRU (Climatic Research Units)[20], and ERA5 (ECMWF Reanalysis 5th Generation)[21]. As shown in Supplementary Figs. 1 and 2, the model can simulates the basic position of the rain band over South Asia and the Tibetan Plateau. Owing to the significant topographic effects of the Tibetan Plateau, high precipitation is located along the windward slopes of the Himalayas. Precipitation is observed and simulated decreasing from south to north. Compared with the reanalysis datasets, the simulated precipitation showed the effects of the terrain, such as high precipitation along the Himalayas; such are not captured in the interpolated gridded datasets as the in-situ observations, on which they rely, are sparse. As noted by Ji et al[22]., gridded observation data may be unreliable in unpopulated regions. For instance, it is well known that precipitation is heavy on the northern slopes of the Kunlun Mountains, but the gridded precipitation products underestimate the precipitation as the interpolation schemes use stations that lie in the arid and semi-arid areas (e.g., the Tarim Basin and Taklimakan Desert)[23].

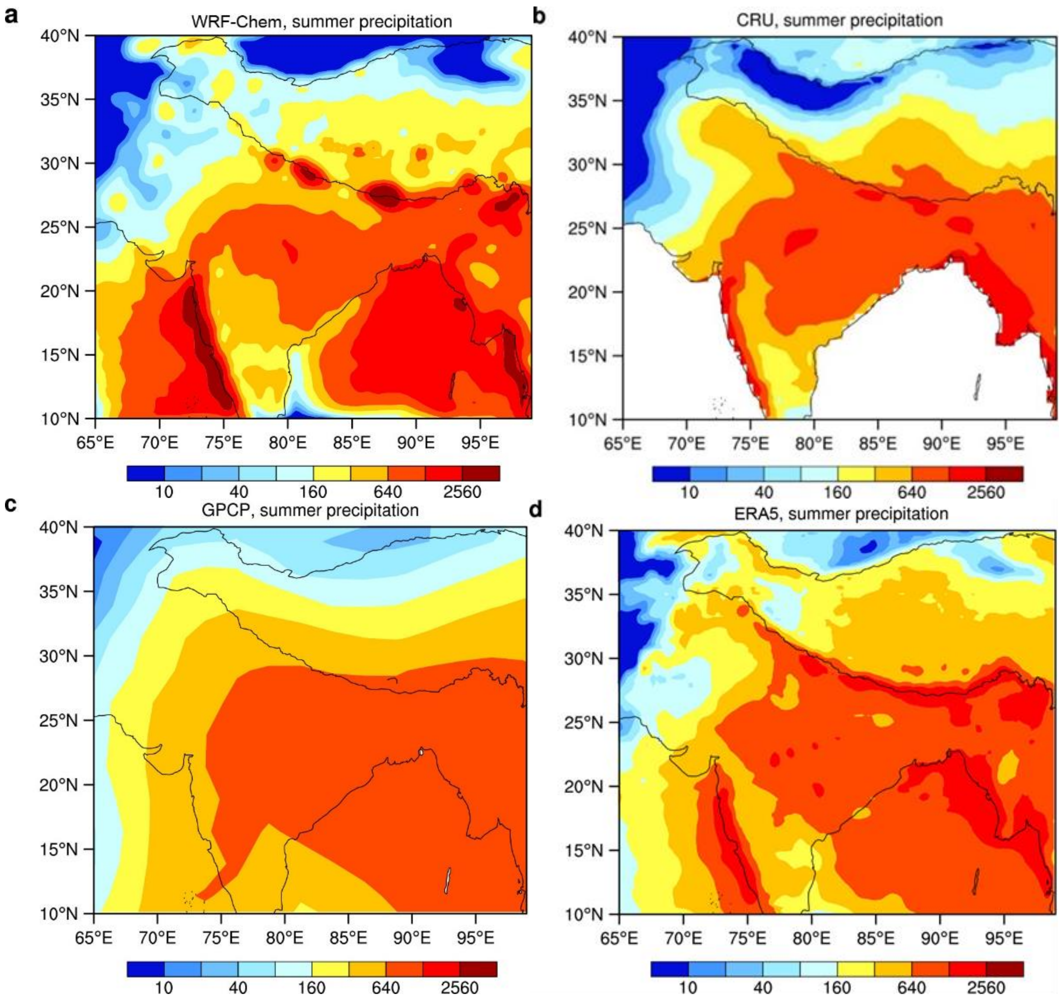

Supplementary Fig. 1 Comparisons between WRF-Chem simulations and reanalysis datasets. a WRF-Chem simulated 2016 summer precipitation (mm), as well as **b** CRU, **c** GPCP, and (d) ERA5 observations of summer precipitation (mm) in 2016.

Supplementary Fig. 2 shows the impact of the cumulus parameterization schemes on summer precipitation is significant. The three panels in Supplementary Fig. 2 represent the Kain-Fritsch, the Betts-Miller-Janjic, and the GD ensemble cumulus schemes, respectively. Compared with the Kain-Fritsch scheme (Supplementary Fig. 2a–d), the simulation ensemble with Betts-Miller-Janjic scheme (BM, Supplementary Fig. 2e–f) predicts larger summer precipitation over the Tibetan Plateau, especially over the northern part. However, the simulations of the GD ensemble cumulus scheme (Supplementary Fig. 2i–l) have a spatial precipitation pattern similar to the simulations of the Kain-Fritsch scheme (Supplementary Fig. 2a–d), except with less precipitation over South Asia (Supplementary Figs. 2i and 2l) after the combination of

YSU planetary boundary layer scheme. The impact of planetary boundary layer schemes on precipitation is smaller than that of the cumulus parameterizations, and the impact is mainly concentrated over South Asia. South Asian summer precipitation prediction using the YSU boundary layer scheme (the first and last rows in Supplementary Fig. 2) is clearly different from that produced by the MYJ boundary layer scheme (the second and third rows in Supplementary Fig. 2). The least impact on summer precipitation is caused by the choice of the microphysical scheme. The intensity and spatial pattern of precipitation are consistent, by comparing the first row vs. the last row, and second row vs. third row in Supplementary Fig. 2, respectively.

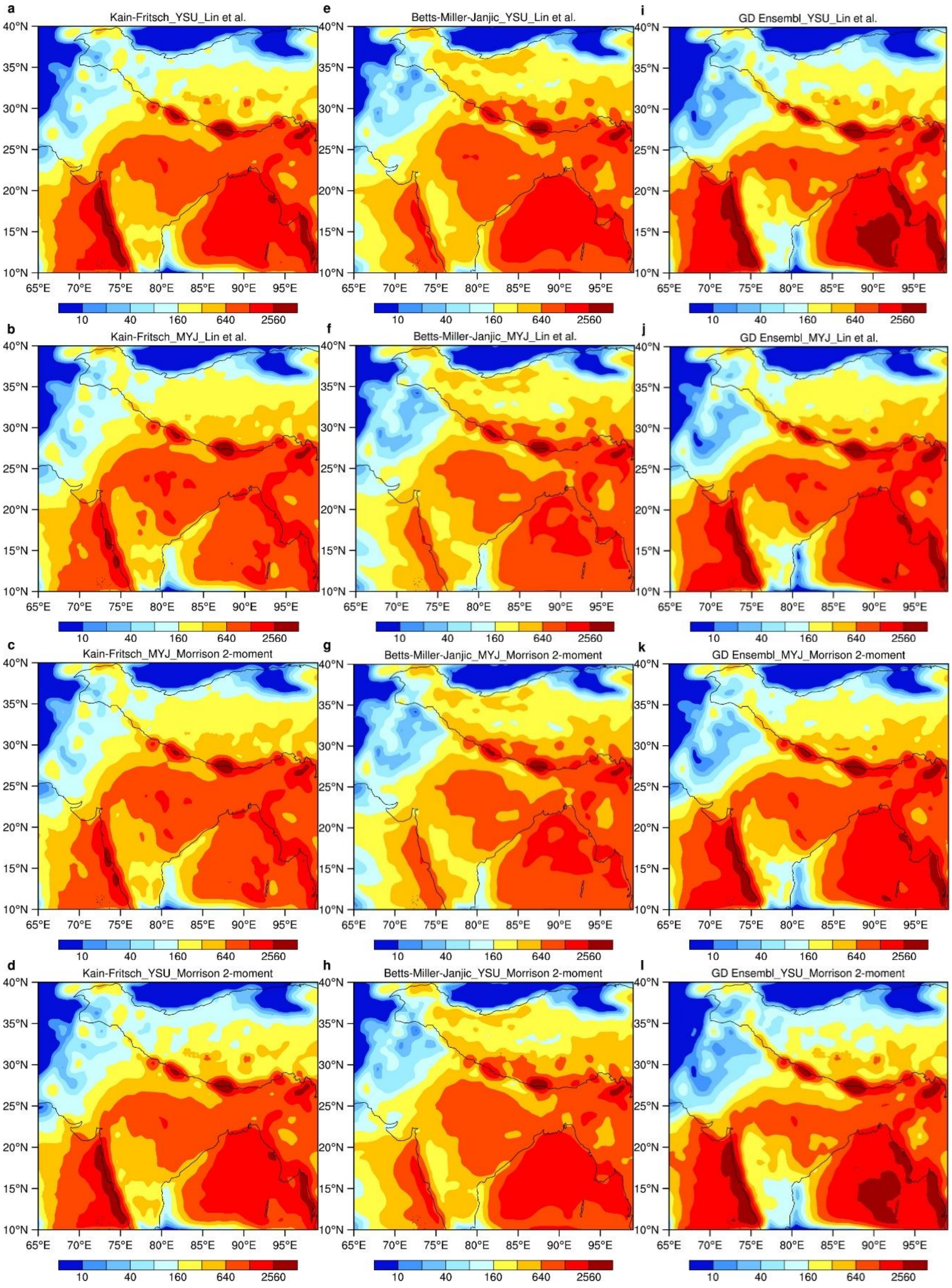

Supplementary Fig. 2 WRF-Chem simulated spatial summer precipitation in 2016 for differing physical parameterization. a–d Kain-Fritsch, **e–h** Betts-Miller-Janjic, and **i–l** GD ensemble cumulus parameterization, microphysical options (Lin et al. scheme (first and second rows) vs. Morrison 2-moment (third and last rows)), and planetary boundary layer option (YSU (first and last rows) vs. MYJ (second and third rows)).

In order to validate WRF-Chem simulation performance, summer precipitation observations from 117 stations in South Asia and 87 stations (Supplementary Fig. 3) were compared with WRF-Chem precipitation estimates.

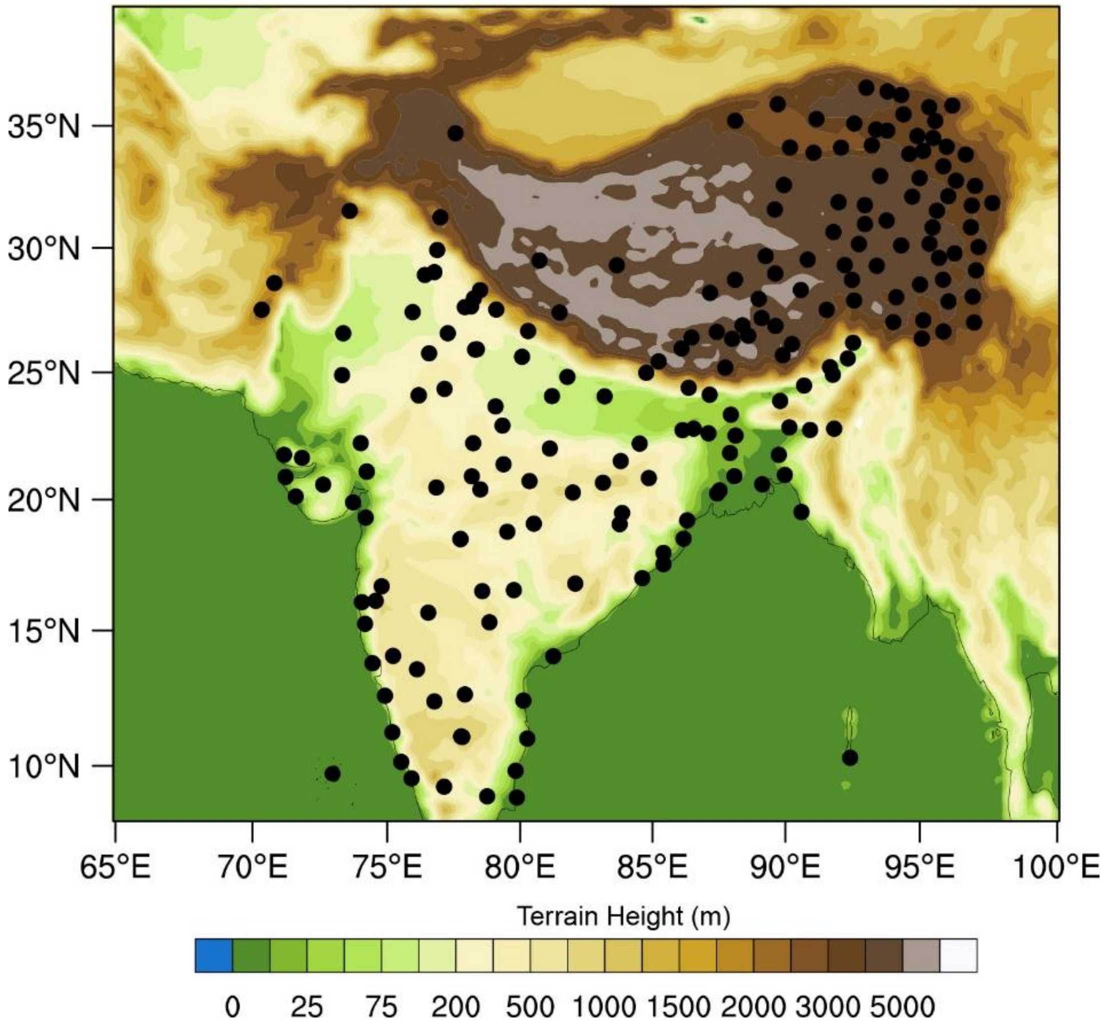

Supplementary Fig. 3 The terrain height and location of in-situ observations for summer precipitation observations (mark with black dots) in the study area.

As listed in Supplementary Table 4, the performance of the ensemble members of WRF-Chem configuration indicate variability: in South Asia, the ensemble members that used the Kain-Fritsch cumulus scheme demonstrated lower ME and RMSE, hence lower bias when compared to the members that used the other two types of cumulus schemes. The Betts-Miller-Janjic cumulus scheme has the greatest bias. Summer precipitation over the Tibetan Plateau was improved by the ensemble members that used the Kain-Fritsch scheme and had greater correlation and lower RMSE when compared to members that used the Betts-Miller-Janjic and the Grell–

Devenyi ensemble cumulus schemes. The Kain-Fristch scheme demonstrated better performance because it considers the scale-aware parameterized cloud dynamics, including subgrid-scale cloud–radiation interactions, a dynamic adjustment time scale, impacts of cloud updraft mass fluxes on grid-scale vertical velocity, and lifting condensation levels based entrainment methodology[24]. Moreover, the aerosol-cloud-radiation interaction is included in WRF-Chem. Therefore, the ensemble members using the Kain-Fristch scheme represent better the formation of precipitation under atmospheric aerosol loading.

Supplementary Table 4 Evaluation metrics for summer precipitation for the various WRF-Chem configurations with in-situ observations at 204 stations in the study area.

	Model setup	R	ME (mm)	MAE (mm)	RMSE (mm)	TMP
South Asia	KF-MYJ-Lin	0.80	61.7	208.7	274.3	2.91
	KF-MYJ-Mor	0.79	33.7	202.8	267.9	2.89
	KF-YSU-Lin	0.82	16.6	212.3	283.5	3.11
	KF-YSU-Mor	0.83	12.5	210.1	251.6	3.01
	BM-MYJ-Lin	0.71	−69.9	241.9	364.2	3.02
	BM-MYJ-Mor	0.69	−101.8	261.3	428.3	3.15
	BM-YSU-Lin	0.72	–66.3	276.6	432.9	3.29
	BM-YSU-Mor	0.73	−58.5	270.6	429.3	3.21
	GD-MYJ-Lin	0.61	110.7	298.3	442.8	2.81
	GD-MYJ-Mor	0.71	108.3	284.6	425.1	2.87
	GD-YSU-Lin	0.64	71.4	295.1	439.8	2.93
	GD-YSU-Mor	0.68	65.9	292.1	433.2	2.97
Tibetan Plateau	KF-MYJ-Lin	0.79	93.5	131.9	391.8	3.87
	KF-MYJ-Mor	0.83	83.1	125.8	213.7	3.24
	KF-YSU-Lin	0.88	10.8	105.1	143.7	2.91
	KF-YSU-Mor	0.86	22.9	109.5	155.6	2.87
	BM-MYJ-Lin	0.74	198.0	230.7	361.2	4.28
	BM-MYJ-Mor	0.72	207.1	242.3	376.6	4.31
	BM-YSU-Lin	0.69	185.0	224.5	352.7	4.03
	BM-YSU-Mor	0.70	198.0	230.7	361.2	4.09
	GD-MYJ-Lin	0.69	117.8	153.3	203.3	3.02
	GD-MYJ-Mor	0.68	103.0	145.5	194.7	2.95
	GD-YSU-Lin	0.65	16.3	108.7	146.6	2.63
	GD-YSU-Mor	0.65	30.1	114.5	160.7	2.68

*KF and BM represent Kain-Fritsch and Betts-Miller-Janjic cumulus parameterizations, respectively. Lin and Mor represent Morrison 2-moment and Lin et al. microphysical options, respectively.

Next, we investigated the sensitivity of each physics scheme to scrutinize bias in the simulated results; this was done by grouping all ensemble members together with a common physical option and comparing their respective bias range using box and whisker plots (Supplementary Fig. 4). The analysis was conducted in the study area using the summer precipitation results from the WRF-Chem simulation along with station observations. The ensemble members with the Kain-Fristch scheme gave the least bias over both South Asia (Supplementary Fig. 4a) and the Tibetan Plateau (Supplementary Fig. 4b). In contrast, the Grell–Devenyi ensemble and Betts-Miller-Janjic cumulus options produced the highest bias. The YSU option when used as the planetary boundary layer scheme tends to produce relatively low levels of bias for both South Asia (Supplementary Fig. 4c) and the Tibetan Plateau (Supplementary Fig. 4d). The Morrison 2-moment scheme as the microphysical option produced the lower bias, compared to the Lin et al. scheme over South Asia (Supplementary Fig. 4e) but, exhibited somewhat higher bias over the Tibetan Plateau (Supplementary Fig. 4f).

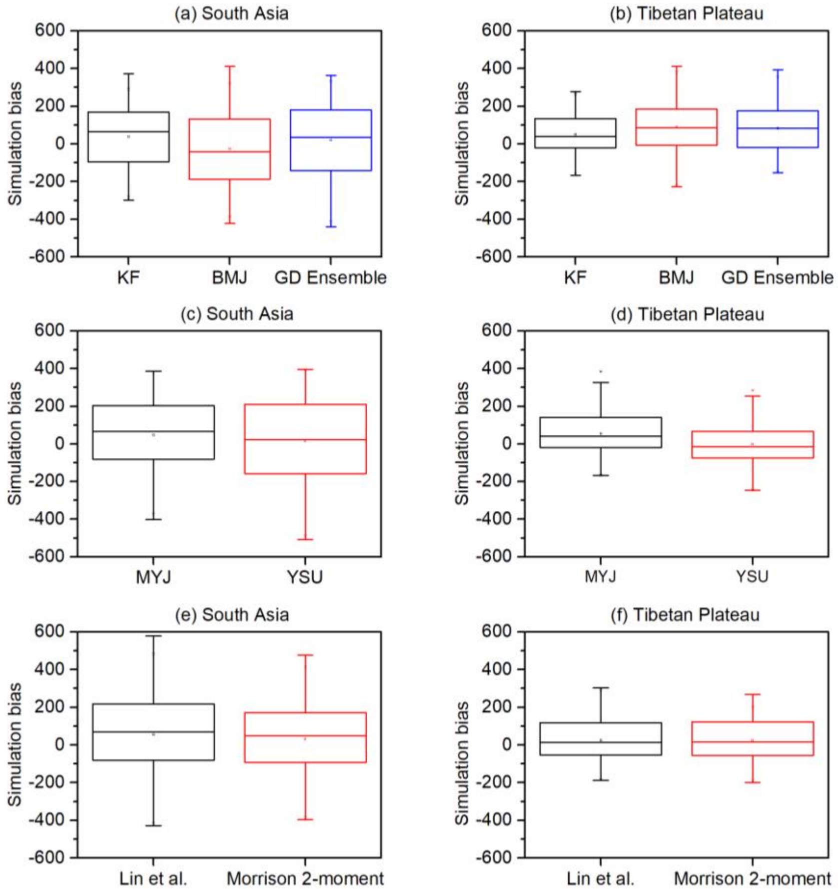

Supplementary Fig. 4 Box and whisker plots representing simulation bias in summer precipitation (mm) for physic schemes. (a) vs. (b) compare the performance for different

cumulus schemes. (c) vs. (d) and (e) vs. (f) compare the performance respectively for each of the planetary boundary layer schemes and microphysics. KF and BMJ demote the Kain-Fritsch and Betts–Miller–Janjic cumulus schemes. The five data points are used to construct the box and whisker plot: the least and greatest values (the whiskers), the median (the middle line), and the quartiles (the ends of the boxes).

The optimal physics scheme combination was identified by considering the correlation coefficient, standard deviation, and RMSE. The combination (Kain-Fritsch cumulus scheme, YSU planetary boundary layer scheme, Lin et al. microphysics scheme) performed best for summer precipitation over South Asia (Supplementary Fig. 5a), while the ensemble member using the Kain-Fritsch cumulus scheme, YSU planetary boundary layer scheme, and Morrison 2–moment microphysics scheme performed the best over the Tibetan Plateau (Supplementary Fig. 5b). In the whole study area, the ensemble member that used the Kain-Fritsch cumulus scheme, YSU planetary boundary layer scheme, Lin et al. microphysical scheme performed the best (Supplementary Fig. 5c). By using the Kain-Fritsch cumulus scheme and the YSU planetary boundary layer, the ensemble members performed equally well for both South Asia and the Tibetan Plateau and the whole study region with correlation values of more than 0.75. Although both the Lin et al. and Morrison 2–moment microphysical scheme are able to represent the aerosol indirect effect (chemistry-chemistry interactions), the ensemble member that used the Lin et al. microphysics scheme with the YSU and Kain-Fritsch cumulus schemes had the highest simulation accuracy, hence this combination was selected.

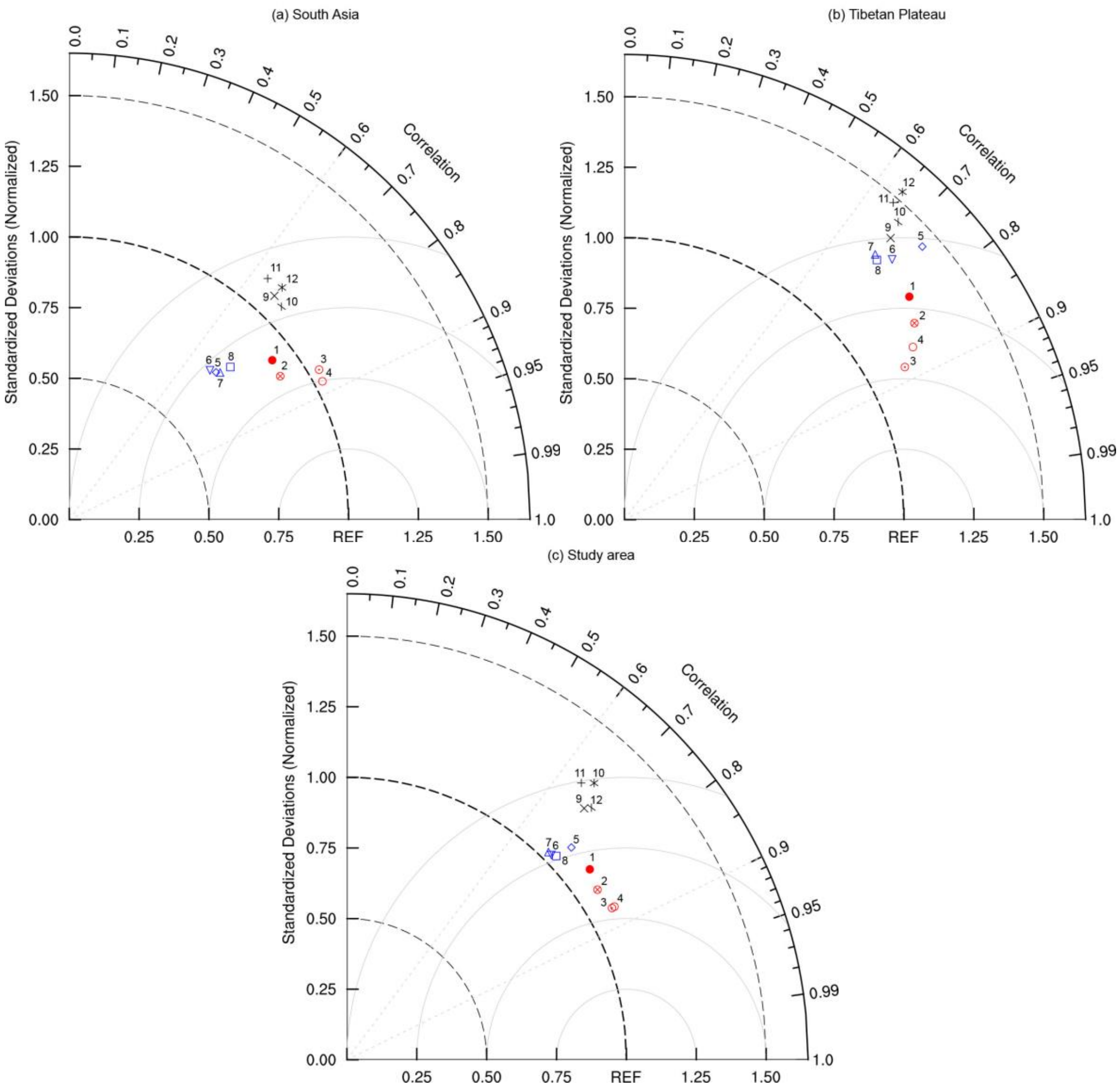

Supplementary Fig. 5 Taylor diagrams with standard deviations and correlation coefficients for summer precipitation for each ensemble member used to evaluate the model performance. (a) South Asia, (b) Tibetan Plateau, and (c) Study area. The ensemble member 1 to 12 represent different configurations, i.e., 1: KF-MYJ-Lin, 2: KF-MYJ-Mor, 3: KF-YSU-Lin, 4: KF-YSU-Mor, 5: BM-MYJ-Lin, 6: BM-MYJ-Mor, 7: BM-YSU-Lin, 8: BM-YSU-Mor, 9: GD-MYJ-Lin, 10:GD-MYJ-Mor, 11: GD-YSU-Lin, 12: GD-YSU-Mor. KF and BM represent Kain-Fritsch and Betts-Miller-Janjic cumulus parameterizations, respectively. Lin and Mor represent Lin et al. and Morrison 2-moment microphysical options, respectively.

Finally, we compared the simulation biases to observations of precipitation. As noted previously, simulation similarity with gridded precipitation (Supplementary Fig. 1) characterized well the basic position of the rain band over South Asia and the Tibetan Plateau. However, the WRF-Chem simulated precipitation showed the effect

of subtle orographic features, i.e., high precipitation along the Himalayas. In our previous use of WRF-Chem[25], comparison with observed precipitation from 73 national meteorological monitoring stations over the Tibetan Plateau showed that the simulations adequately captured the seasonal variation of precipitation but, with an underestimation from May to October. In this study, the previous underestimation of precipitation was improved upon as was shown in Supplementary Table 4; this is probably due to the use of the Kain-Fristch cumulus scheme rather than the Grell–Devenyi Ensemble cumulus scheme used previously. The Kain-Fristch scheme demonstrated improved precipitation simulation for the reasons articulated earlier.

1.3.3 Evaluation of black carbon

In previous work, we had confirmed that the CBMZ gas-phase chemical mechanism and MOSAIC aerosol module were the most suitable in the reconstruction of black carbon (BC) concentrations over the Tibetan Plateau and adjacent regions[25,26]. As is shown in Supplementary Table 5, we had compared model simulations of black carbon concentration with in-situ stations from 14 stations. The WRF-Chem model with the aforementioned combination of chemical schemes reproduced well the spatial variation of BC concentrations at these sites[26]. Both simulations and observations reveal low BC concentration levels occurred at sites over the Tibetan Plateau such as Namco, Lhasa, and Qomalangma, with concentrations less than 1 μg m^{-3}, whereas high black carbon levels appeared in highly populated cities such as Xi'an (8.7 μg m^{-3}).

Supplementary Table 5 Observed and simulated surface black carbon concentration (μg m^{-3}) for specified time periods.

Name	Location (N, E)	Time (LT)	Observation	Simulation	
Xi'an	34.2N 108.9E	May 2013–Apr 2014	8.7	11.8	Li et al[27].
Lanzhou	36.0N 103.E	Sep 2010–Aug 2011	7.58	4.51	Li et al[28].
Urumqi	43.8N 87.6E	Nov 2009–Feb 2010	6.15	1.94	Liu et al[29].
Lhasa	29.6N 91.0E	May 2013–Mar 2014	0.14	0.19	Li et al[30].
Qomalangma	28.3N 86.9E	May 2015–Apr 2016	0.29	0.18	Chen et al[31].
Namco	30.7N 90.9E	Jan 2012–Dec 2012	0.19	0.10	Wan et al[32].
Qinghai Lake	36.9N 99.9E	Jan 2012–Dec 2012	0.84	0.58	Zhao et al[33].
Laohugou	39.5N 96.5E	May 2009–Mar 2011	0.48	0.24	Zhao et al[34].
Ranwu	29.3N 96.9E	Nov 2012–Jun 2013	0.14	0.21	Wang et al[35].
Muztagh Ata	38.2N 75.0E	Dec 2003–Dec 2006	0.06	0.11	Cao et al[36].
NCO-P	27.9N 86.8E	Dec 2006–Feb 2008	0.17	0.25	Marinoni et al[37].
Manora Peak	29.4N 79.5E	Feb 2005–Jul 2008	1.14	1.43	Ram et al[38].
Dhaka	23.7N 90.3E	Mar 2010–Feb 2011	22.8	7.81	Begum et al[39].
Sinhagad Pune	18.3N 73.7E	Jan 2010–Dec 2010	3.8	2.7	Safai et al[40].

An additional reanalysis dataset (the Modern-Era Retrospective analysis for Research and Applications version 2 (MERRA-2)) was also used as a comparative for WRF-Chem simulation output; this can be seen in Supplementary Fig. 6. Both WRF-Chem and the MERRA-2 reanalysis characterized high black carbon concentration levels along the Indo-Gangetic Plain with some “hotspots”; this is expected given the intense local anthropogenic emissions there. Comparatively, the Tibetan Plateau exhibits lower concentrations with marginally higher values along the southern edge of the plateau. The WRF-Chem model are more pronounced in orographic regions like the inland regions of the Tibetan Plateau. The detail of the

WRF-Chem simulations is expected due to the higher resolution of WRF-Chem which captures the niceties of topography and its effects on atmospheric dynamics. As reported in a recent study[41], the relatively more detailed topographic resolution used by the WRF-Chem model resulted in 50% more black carbon transport across the Himalayas.

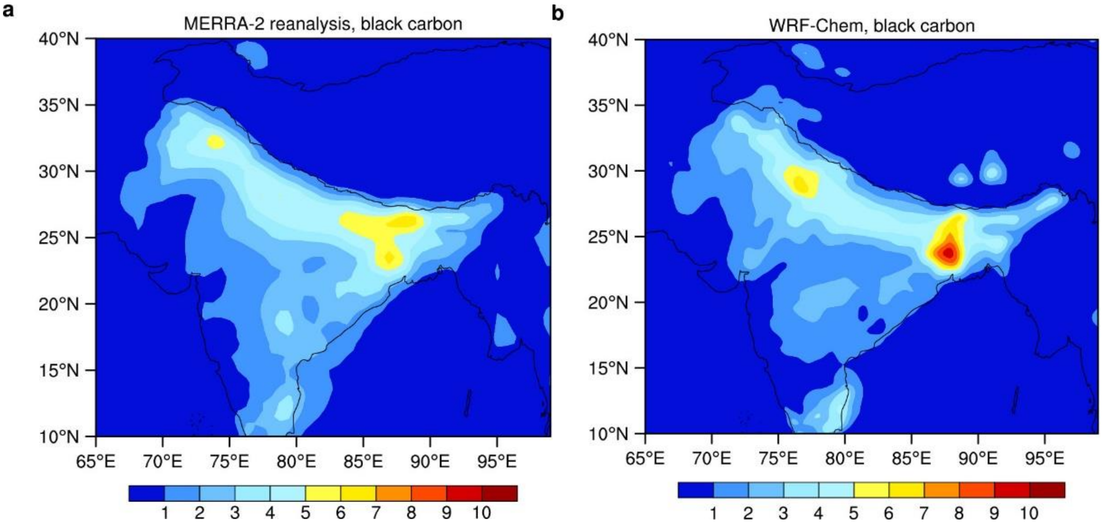

Supplementary Fig. 6 Surface 2016 surface black carbon concentration values (μg m^{-3}). **a** MERRA-2 reanalysis and **b** WRF-Chem simulation.

We further evaluate the WRF-Chem model performance in black carbon simulation by utilizing in-situ observations from the coordinated monitoring and research network of atmospheric pollution and cryospheric changes (APCC)[42] to evaluate the model performance in black carbon simulation. The APCC was initiated by our group in 2013 to conduct a more integrated and in-depth investigation of the origins and distribution of atmospheric pollutants and their effects on cryospheric change, covering the Tibetan Plateau and the surrounding region (Supplementary Fig. 7). The APCC measurement network currently consists of 30 stations that collect samples from the atmosphere, glaciers, and snow cover.

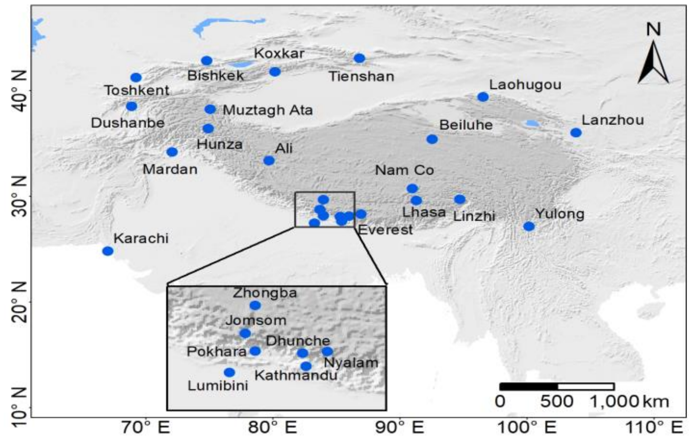

Supplementary Fig. 7 The atmospheric stations location of the APCC network referring to Kang et al[42]. The base map image is plotted by the author Shichang Kang based on the downloaded Digital Elevation Model (DEM) data from the open source website (http://www.gscloud.cn/sources/?cdataid=302&pdataid=10).

Supplementary Table 6 identified the 20 meteorological (atmospheric) stations in the study area. Supplementary Fig. 8 shows the mean and standard deviation of measured and modeled black carbon concentrations from the 20 atmospheric sites. The model simulated values of black carbon were comparable to in-situ observations at the majority of atmospheric sites. Specifically, the simulated black carbon concentrations show high values in urban sites such as Lanzhou (6.96 ± 3.56 μg m^{-3}) and Kathmandu (5.7 ± 2.73 μg m^{-3}), followed by Dushanbe (3.89 ± 1.51 μg m^{-3}) and Mardan (4.32 ± 2.89 μg m^{-3}). In contrast, relatively low surface black carbon concentrations are found in the remote sites, such as Nam Co (0.32 ± 0.28 μg m^{-3}), Everest (0.44 ± 0.23 μg m^{-3}), and Lhasa (0.97 ± 0.42 μg m^{-3}). Overall, the model represented well the black carbon concentration at these sites well over the simulation period (Supplementary Fig. 8).

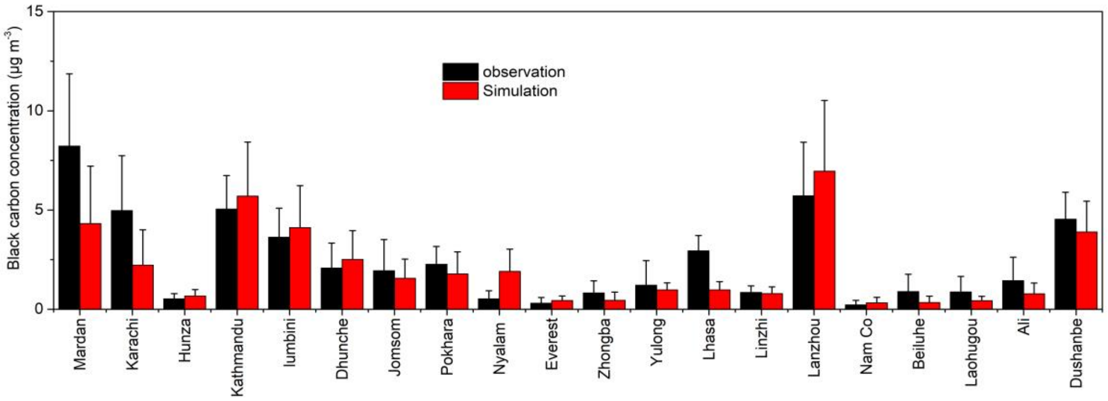

Supplementary Fig. 8 Comparison of measured and simulated average black carbon concentration at stations from APCC network. The whiskers represent the greatest values.

Supplementary Table 6 Detailed geographic information of APCC meteorological sites over the Tibetan Plateau surrounding area. (*masl* is meters above sea level)

	Full station name	Location	Elevation (*masl*)
Ali	Ngari Station for Desert Environment Observation and Research, western Tibetan Plateau	33.4N, 79.7E	4270
Laohugou	Qilian Observation and Research Station of Cryosphere and Ecologic Environment, northern Tibetan Plateau	39.4N, 96.5E	4230
Beiluhe	Beiluhe Observation and Research Station in Qinghai-Tibet Plateau	35.4N, 92.5E	4000
Namco	Nam Co Station for Multisphere Observation and Research, southern Tibetan Plateau	30.8°N, 91.0E	4730
Nyalam	Nyalam, southern Tibetan Plateau	28.2N, 86.0E	4166
Everest	Qomolangma Atmospheric and Environmental Observation and Research Station (Everest), Himalayas	28.3N, 86.9E	4276
Linzhi	southeastern Tibetan Plateau	29.7N, 94.7E	3326
Yulong	Yulong Snow Mountain Glacial and Environmental Observation and Research Station	27.2N, 100.2E	2650
Lanzhou	Lanzhou city, Gansu Province	36.0N, 103.9E	1520
Lhasa	Lhasa city, Xizang (Tibet) Autonomous Region	29.6N, 91.3E	3642
Dhunche	Dhunche	28.1N, 85.3E	2051
Pokhara	Pokhara	28.2N, 84.0E	813
Jomsom	Jomsom	28.8N, 83.7E	3048
Kathmandu	Kathmandu	27.7N, 85.4E	1300
Lumbini	Lumbini	27.5N, 83.3E	100
Karachi	Karachi	24.9N, 67.0E	13
Mardan	Mardan	34.2N, 72.0E	485
Dushanbe	Dushanbe	38.5N, 68.9E	864
Hunza	Hunza	36.5N, 74.9E	2519
Zhongba	Zhongba, southern Tibetan Plateau	29.7N, 84.0E	4704

As to model evaluation metrics for 20 APCC stations, these are highlighted in Supplementary Fig. 9. Additionally, Supplementary Fig. 10 is a plot of simulated and observed daily mean black carbon concentrations at the Qomolangma (Mt. Everest) station (QOMS, 28.36N, 86.95E, 4276 *masl*). It appears that the WRF-Chem tends to underestimate the black carbon in high spike conditions but does reproduce a reasonable seasonal profile.

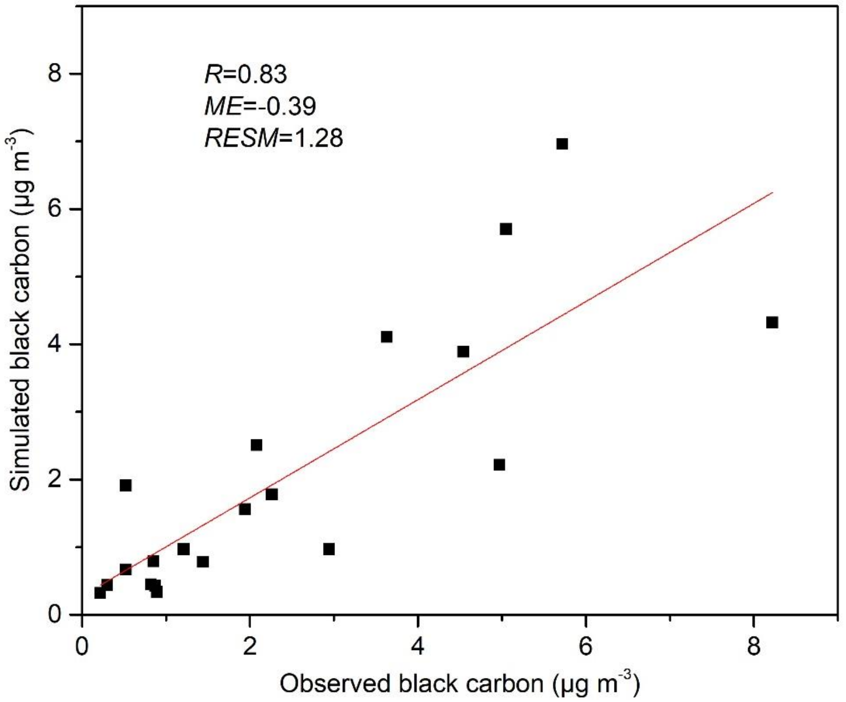

Supplementary Fig. 9 Scatter diagram for measured and simulated average black carbon concentrations at APCC stations.

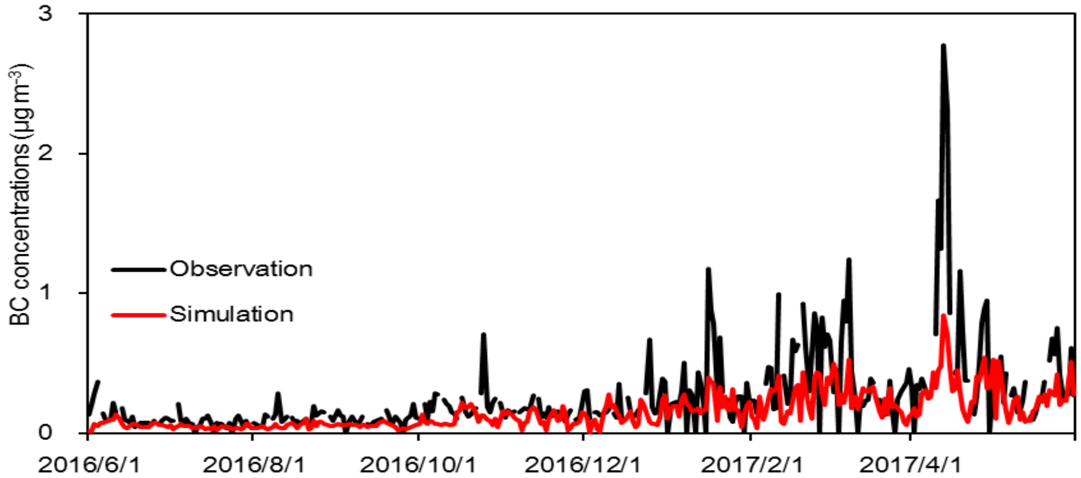

Supplementary Fig. 10 The simulated and observed daily mean black carbon concentrations at the QOMS station.

A further evaluation of the WRF-Chem performance was undertaken at the event scale; this was for four pollution incidents where black carbon exceeded 1 μg m^{-3} at QOMS. The four instances were (i) incident A – June 8–10, 2015, (ii) incident B – March 19–22, 2016, (iii) incident C – April 9–18, 2016, (iv) incident D – April 11–14, 2017. Supplementary Fig. 11 details all of the four pollution episodes. The

WRF-Chem model captured the variations in trend of observed surface black carbon concentrations – correlation coefficients were all above 0.8, implying that the model was able to reproduce black carbon concentration distributions. It is also noteworthy to mention that black carbon underestimation by the chemical transport model. This is, at least in part, because the model grid is a point source regional average computation over 25km, whereas the observation at QOMS site is more strongly influenced by complex local topography that is not represented well in the model due to the model's coarse resolution[43,44,45]. Model discrepancies also likely originate from underlying uncertainties such as inadequate representation of emissions data and the representation of the planetary boundary layer height[46,47,48]. As noted earlier, we updated the initial chemical conditions with representative values and selected the optimal combination of physical schemes. Therefore, when compared with in-situ observation data, the model reproduced satisfactory results of seasonal surface black carbon as well as capturing their spatial variability.

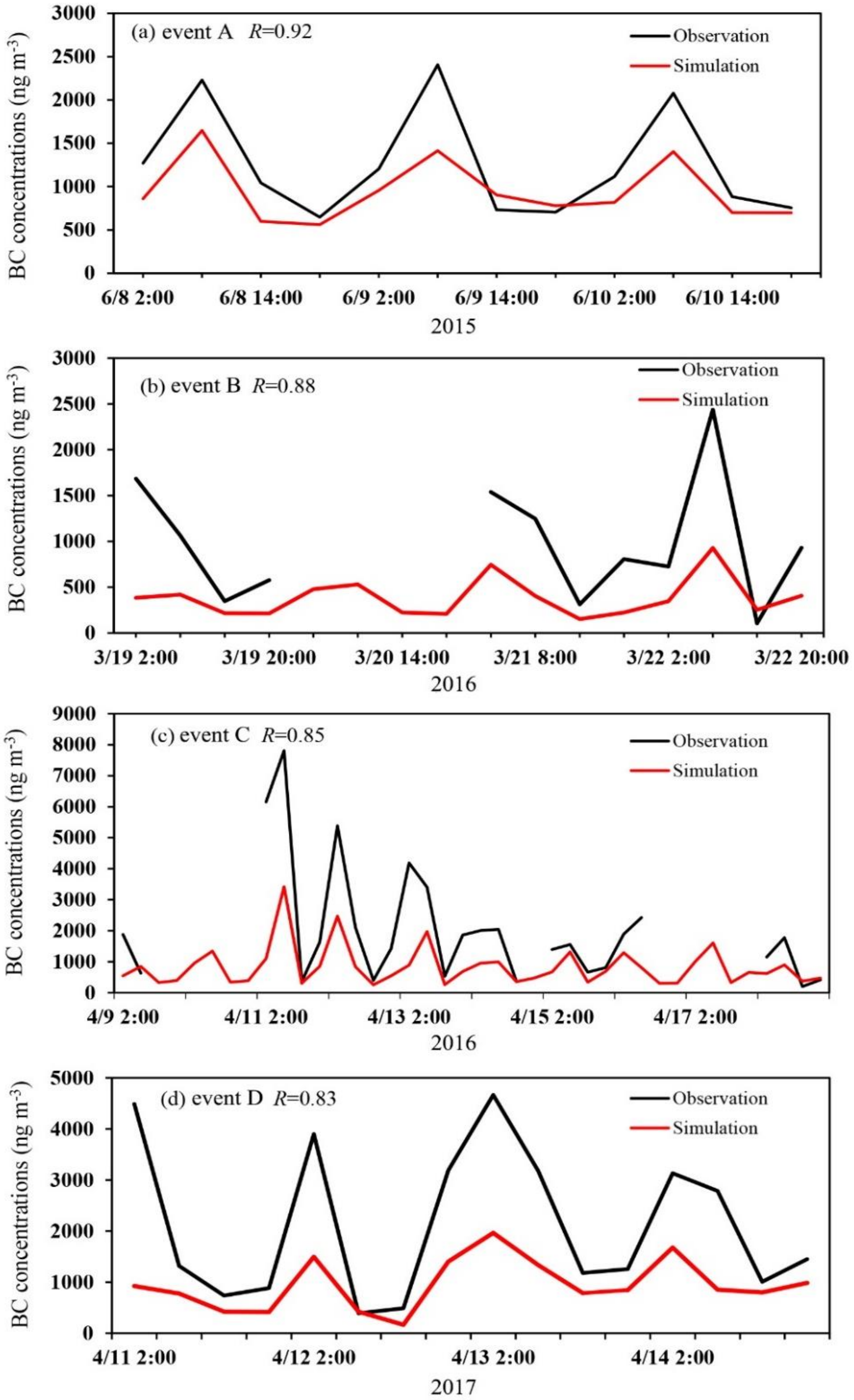

Supplementary Fig. 11 Comparisons between simulated black carbon (BC) concentrations and observations at QOMS for the four pollution episodes. **a** event A, **b** event B, **c** event C, and **d** event D.

1.3.4 Model uncertainty analysis

In addition to different physical and chemical schemes, variations in spatial resolutions can also result in simulation uncertainties. In this study, we conducted the WRF-Chem simulations at a 25 km spatial resolution. To analyze model uncertainty caused by the spatial resolution, we designed a higher resolution WRF-Chem simulation, i.e., at a resolution of 8 km – the result can be seen in Supplementary Fig. 12. A comparison of both model resolution runs of the 500 hPa wind field with the ERA-Interim reanalysis is plotted in Supplementary Fig. 12a and confirms that each resolution captures the essence of summer dynamics, showing a local disturbance in the Indian subcontinent and southerly winds prevailing in the southern Tibetan Plateau. Additionally, we compared the vertical velocity simulated along 20°N. Supplementary Fig. 13, modelled vertical velocity, indicates that the 25-km spatial resolution simulation was just as representative as that at the 8-km resolution. Therefore, we concluded that a resolution of 25 km was adequate to capture local convection in both the horizontal and vertical dimensions.

Moreover, reference to previous studies was invaluable in our choice of resolution. Global climate models (GCMs), with low horizontal resolution have been used to study the effect of black carbon on the South Asian monsoon and the precipitation; these included CESM[49] (1.9° (latitude) × 2.5° (longitude)), PCM[50] (≈ 300km), CCM3[51] (T42/L18), NASA finite-volume GCM[52,53]. Given the GCMs low resolution, subtle characteristics of atmospheric dynamics in complex terrain cannot be adequately resolved. Additionally, high-resolution regional climate models (RCMs) have been used: Ji et al[23]. used the RegCM-chemistry model to investigate the effects of black carbon on the Indian summer monsoon at 50 km resolution in the horizontal. Soni et al[54]. used the WRF-Chem model (at 30 km horizontal resolution) and found lower tropospheric heating by black carbon caused increased convection and rainfall in North-East India during the pre-monsoon season. Likewise, Dong et al[55]. successfully used the WRF model at a 27 km horizontal resolution to simulate the deep convection over the Indian subcontinent at precipitation event scales. Given the abilities of the RCMs to capture local convection over South Asia at coarser resolutions, we felt justified that our 25 km would resolve the key climate dynamics of interest.

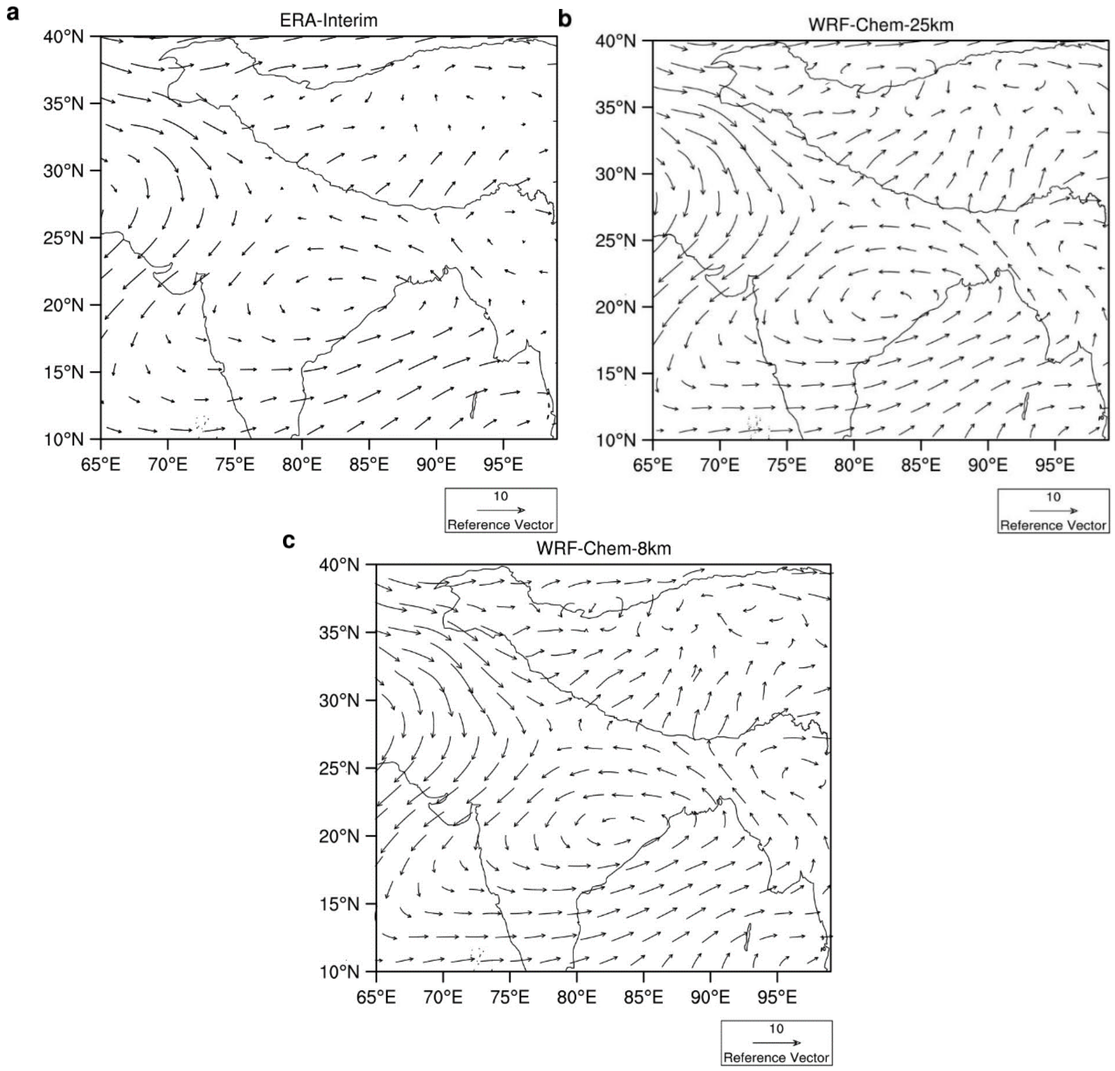

Supplementary Fig. 12 Summer 500 hPa wind (units: ms^{-1}) in 2016. a the ERA-interim re-analysis and, WRF-Chem simulations with different spatial resolutions (**b** 25km & **c** 8km).

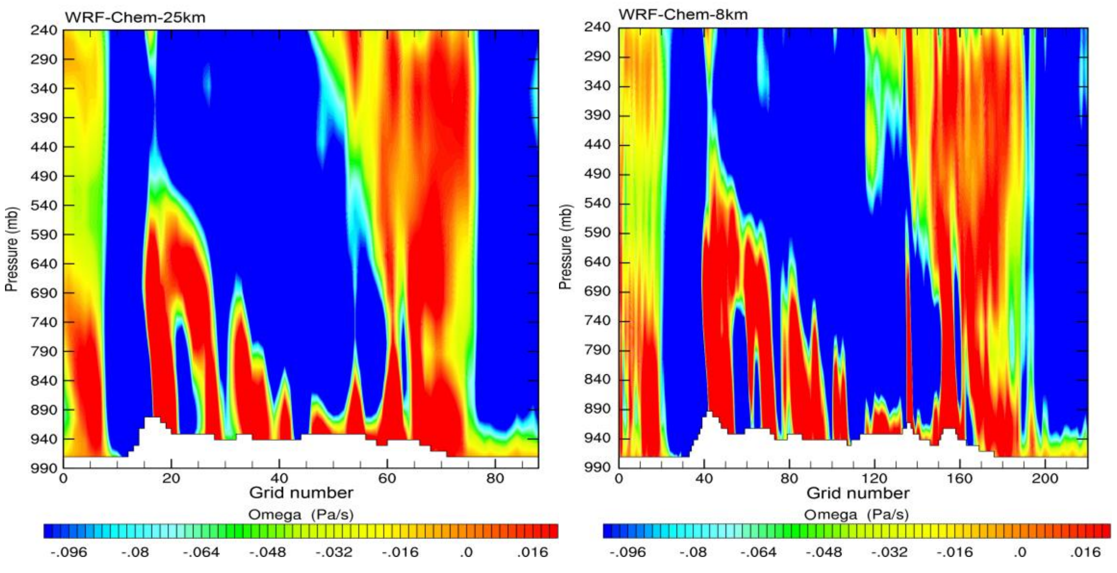

Supplementary Fig. 13 Summer vertical velocity (omega: Pa/s) in 2016 from WRF-Chem simulations along 20°N at different resolutions. a 8 km and **b** 25-km.

Finally, we discuss the ensemble experiments using the Kain-Fristch cumulus scheme; this is shown in Supplementary Fig. 14. The single simulations using the Kain-Fritsch cumulus scheme captured the daily precipitation when compared to a baseline (the ERA5 reanalysis); this emphasizes the value of the ensemble modeling where running two or more related but different analytical models and then synthesizing the results into a single score or spread improves the accuracy of predictive analytics.

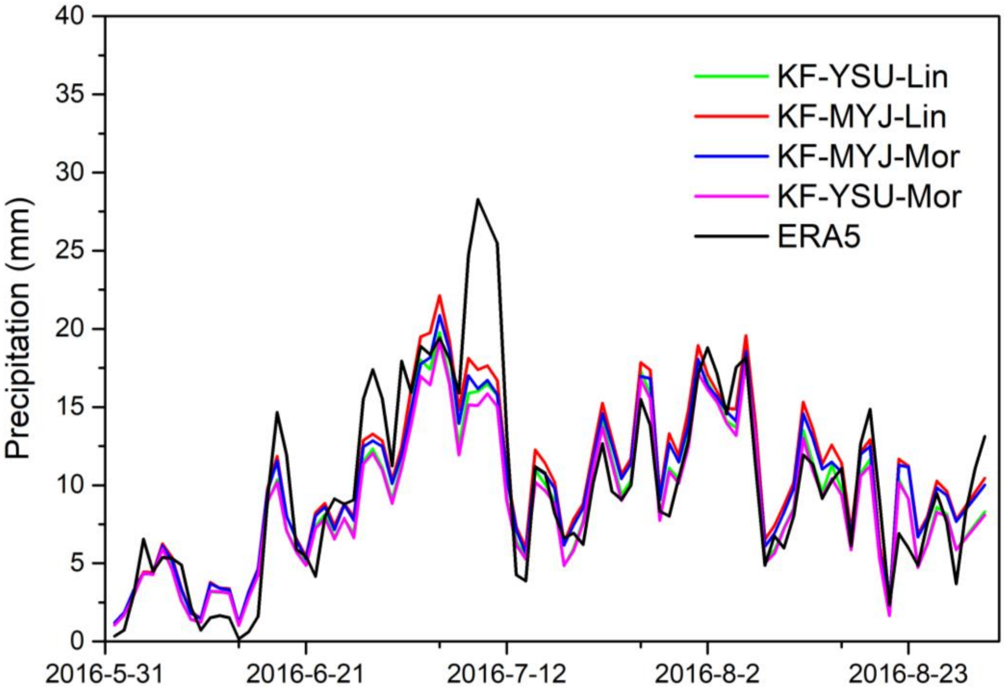

Supplementary Fig. 14 Comparison of daily precipitation from the ensemble WRF-Chem simulations using the Kain-Fritsch cumulus scheme to that from the ERA5 reanalysis data during the simulation period. Scheme abbreviations are given in Supplementary Table 4.

1.4 Glacier mass balance model

The monthly-scale mass balance model from Radić and Hock[56] was used to model glacier mass balance for the Tibetan Plateau for the period 1979–2014. The primary input data in this model includes monthly precipitation and air temperature; this was obtained from the CRU dataset. Glacier area-weighted specific mass balance (B) for the whole glacier in each mountain range was calculated as a sum of the specific mass balance (b) of each elevation band on a glacier (i):

$$B = \frac{\sum_{i=1}^{n} b_i \cdot s_i}{\sum_{i=1}^{n} s_i} \quad (8)$$

where b_i and S_i denote specific mass balance and glacier area, respectively, subscript (i) represents the number of elevation bands on a glacier (i = 1, 2, 3, …, n) with an elevation interval of 50 m.

Monthly specific mass balance (b_i, mm water equivalent) for each elevation band on a glacier was calculated as:

$$b_i = a_i + c_i + R_i \quad (9)$$

where a_i is glacier surface ablation (negative), c_i is glacier mass accumulation (positive), and R_i refers to snowmelt refreezing (positive) at each elevation band.

For debris-free glaciers, the glacier surface ablation (a_i, mm water equivalent) was calculated based upon a positive degree-day model[57] in which snow/ice melt is considered linearly correlated to monthly air temperature. Here, monthly a_i was computed as:

$$a_i = f_{snow/ice} \times \int \max(T_i, 0)\, dt \quad (10)$$

where $f_{\text{snow/ice}}$ denotes the degree-day factor for snow/ice (mm water equivalent day^{-1} °C^{-1}), and T_i denotes monthly air temperature (°C) above the glacier surface.

For debris-covered glaciers, the relationship between debris thickness and glacier surface ablation[58] was used to calculate the effect of debris cover on glacier surface ablation. In detail, an averaged curve for debris thickness and surface ablation was used in the mass balance model to calculate the reduction of ice ablation due to debris cover. To calculate the ablation of debris-covered glaciers, the ablation factor k_i was introduced to quantify the effect of the debris on glacier surface ablation. Thus, monthly glacier surface ablation due to debris cover (a_i, debris) was calculated as:

$$a_{i,\text{debris}} = k_i \times f_{snow/ice} \times \int \max(T_i, 0)\, dt \quad (11)$$

where k_i denotes the scale factor for each elevation band (i) and depends on the debris thickness.

Monthly glacier mass accumulation for each elevation band c_i (mm water equivalent) was calculated as:

$$c_i = \delta_m \times P_i \begin{cases} \delta_m = 1, & T_i < T_{snow} \\ \delta_m = 0, & T_i \geq T_{snow} \end{cases} \quad (12)$$

where δ_m is a constant, T_i denotes air temperature at each elevation band, and T_{snow}

represents the threshold temperature differential between snow and rainfall. If T_i is below T_{snow}, P_i is assumed to be snow. Otherwise, P_i is regarded as liquid precipitation.

Based on the relationship between annual potential refreezing $R_{i,\text{pot}}$ (cm) and the air temperature T_a (°C) at each elevation band[40], $R_{i,\text{pot}}$ was calculated by equation (13):

$$R_{i,\text{pot}} = -0.69 \times T_a + 0.0096 \tag{13}$$

where the lower boundary of annual snowmelt refreezing over the whole glacier is zero, while an upper boundary was applied in the ablation zone and assumed equal to the accumulated snow. Monthly snow meltwater frozen on the glacier surface (R_i) does not flow away until the accumulated melt in a mass balance year exceeds the annual potential refreezing ($R_{i,\text{pot}}$).

In order to interpolate the Climatic Research Unit Time-Series (CRU TS) monthly temperature at each elevation band, we applied two temperature lapse rates (T_lap, and G_lap): T_lap corresponds to the 'statistical lapse rate' between the CRU TS altitude of the grid cell at glacier location (h_CRU) and the highest elevation of the glacier (h_max), while G_lap denotes glacier surface temperature taking into account glacier surface configurations such as orientation and glacier surface climate environment.

Monthly air temperature at each elevation band (T_i) was calculated as:

$$T_i = T_{CRU} + T_\text{lap} \times (h_{max} - h_{CRU}) + G_\text{lap} \times (h - h_{max}) \tag{14}$$

where T_CRU denotes the CRU TS monthly air temperature for the period from 1979 to 2014, and h represents the altitude of the glacier elevation band.

To scale up CRU TS monthly precipitation to h_max, a precipitation correction factor (k_p) was assigned, while a precipitation gradient (d_pre) was used to interpolate precipitation to each elevation band (the percentage of precipitation decreases with every 50 m decrease in elevation) from the highest to lowest elevation of the glacier. Thus, monthly precipitation for each elevation band was calculated as:

$$P_i = k_p \times P_{CRU} \times \lfloor 1 + d_{pre} \times (h - h_\text{max}) \rfloor \tag{15}$$

where P_CRU denotes CRU TS monthly grid precipitation from 1979–2014 at each cell location on the glacier.

The glacier mass balance sensitivities to temperature change ($\Delta B/\Delta T$) and precipitation change ($\Delta B/\Delta T$) were calculated as:

$$\frac{\Delta \acute{B}}{\Delta T} = \Delta \acute{B}(\pm\Delta T) - \Delta \acute{B} \quad (16)$$

$$\frac{\Delta \acute{B}}{\Delta P} = \Delta \acute{B}(\pm\Delta P) - \Delta \acute{B} \quad (17)$$

where B is the simulated annual average mass balance (m water equivalent year^{-1}) for the period from 1979–2014, B (ΔT) is the same B but in response to a step-wise temperature change in the range from −6 K to +6 K incremented by 0.5 K, and B (ΔP) is the same B, but in response to a step-wise precipitation change from −30% P to +30% P with incremented by 5% P: these were advanced in an uniform stepwise change to the original monthly temperature and precipitation time series throughout the 36-year period.

2. Precipitation and incoming moisture variations over the Tibetan Plateau

2.1 Precipitation trends over the Tibetan Plateau

We utilized the GPCP monthly precipitation dataset to investigate the precipitation trend over the Tibetan Plateau. The GPCP is a monthly mean rainfall dataset. GPCP is a merged dataset on a 2.5-degree global grid derived from rain gauge station data, satellite derived data, and sounding observations for the period 1979 to the present. As shown in Supplementary Fig. 15, the GPCP precipitation trend corroborates the increased summer precipitation trend (derived from the CRU dataset) over the southern Tibetan plateau and, more importantly confirms the trend reversal at the beginning of 21st century. Based upon our earlier analysis of CRU monthly gridded precipitation data, it had been established that, in the northern Tibetan plateau, there has been an increase in summer precipitation over the past 56 years.

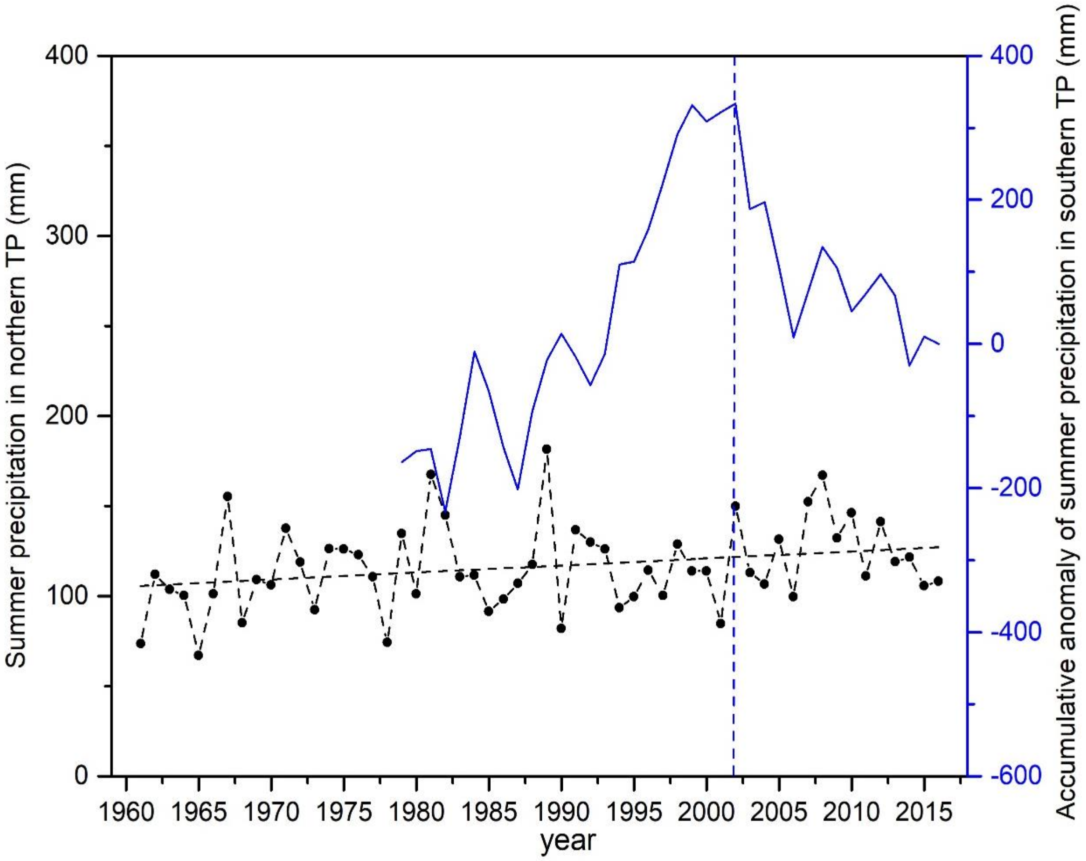

Supplementary Fig. 15 Summer precipitation trend over the Tibetan Plateau (TP). Linear trends in summer area-averaged precipitation over the northern TP using the Climatic Research Unit dataset (period of record (por) 1961–2016, black dashed line) and the accumulative anomaly in summer area-averaged precipitation over southern TP using Global Precipitation Climatology Project (GPCP) dataset (por 1979–2016, blue solid line).

In the meantime, and covering the study domain, a further dataset from the China Meteorology Data Service Centre (CMDSC), which contained daily precipitation data from 86 national observational stations was used to analyze precipitation trends for the 1961–2014 period. The daily analysis likewise confirms the decreasing trend in summer precipitation over the southern Tibetan plateau during 2004–2016; this accompanied by a concomitant increasing trend for the northern Tibetan plateau (Supplementary Fig. 16a). Subsequently, we calculated a time subset (i.e., the 2004–2016 period using CRU) for the summer precipitation trends over the southern Tibetan plateau and adjacent regions as plotted in Supplementary Fig. 16b. As expected, the CRU analysis revealed a decreasing trend in the summer precipitation was evident over the southern Tibetan plateau, while the opposite was the case for many regions within Indian subcontinent.

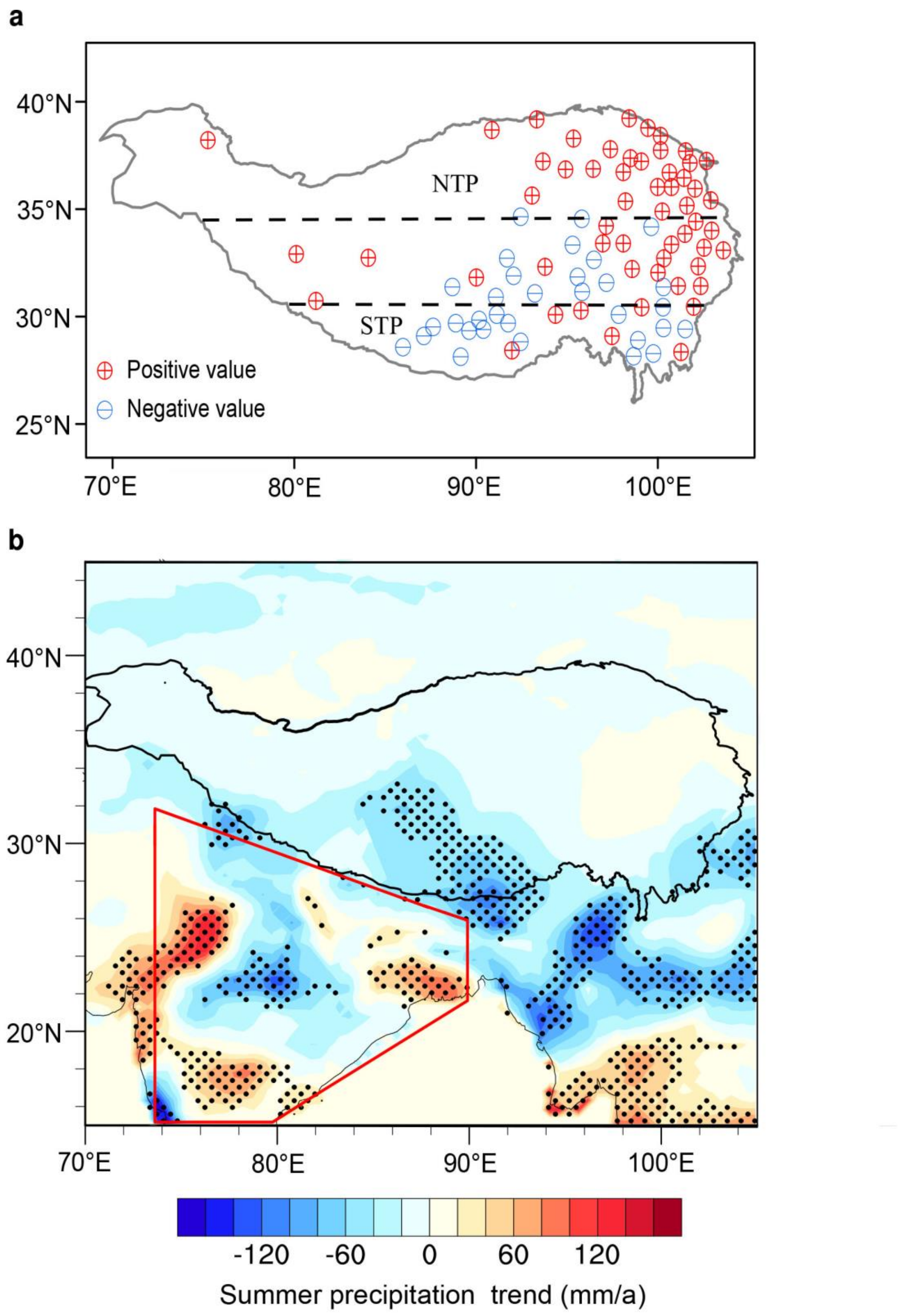

Supplementary Fig. 16 Summer precipitation change. a Summer precipitation trend overv the Tibetan plateau during 2004–2016 using CMDSC in-situ observations. **b** CRU derived summer precipitation trend over the Tibetan plateau and South Asia for period of record 2004–2016 using CRU. NTP and STP in **a** represent the northern Tibetan plateau and the southern Tibetan plateau, respectively. The red polygon in **b** represents South Asia. Black dots in **b** indicate statistically significant areas.

2.2 Change of incoming moisture from South Asia to the southern Tibetan Plateau

In the southern Tibetan plateau, the impact of localized surface evaporation is not particularly pronounced[59], thus the inter-annual variability of the summer precipitation is primarily controlled by “long-range” moisture transport. To investigate moisture transport and moisture change from South Asia to the southern Tibetan Plateau, we computed the moisture flux and its divergence using the European Center for Medium-Range Weather Forecasts (ECMWF) Interim Reanalysis (ERA-Interim) for the 1989–2018 period; the analysis is plotted in Supplementary Fig. 17a. Supplementary Fig. 17a indicates a predominance of negative divergent values of summer moisture flux over South Asia and the southern Tibetan Plateau; this implies convergent zones of water vapor for the aforementioned regions. Moreover, the water vapor flux field substantiates moisture source over southern Tibetan Plateau is one of long-distance transport from South Asia. As for change at the “decadal” scale (Supplementary Fig. 17b), increased summer transport of moisture flux change indicates that incoming water vapor flux was reduced during the 2004–2018 period in the southern Tibetan Plateau compared with the prior period (i.e., 1989–2003). However, Supplementary Fig. 17b reveals that, summer divergence of moisture flux was reduced for South Asia during 2004–2018 and, there was a prominent strengthening of the cyclonic circulation in South Asia, the consequence being more water vapor flux convergence in the latter “decade”.

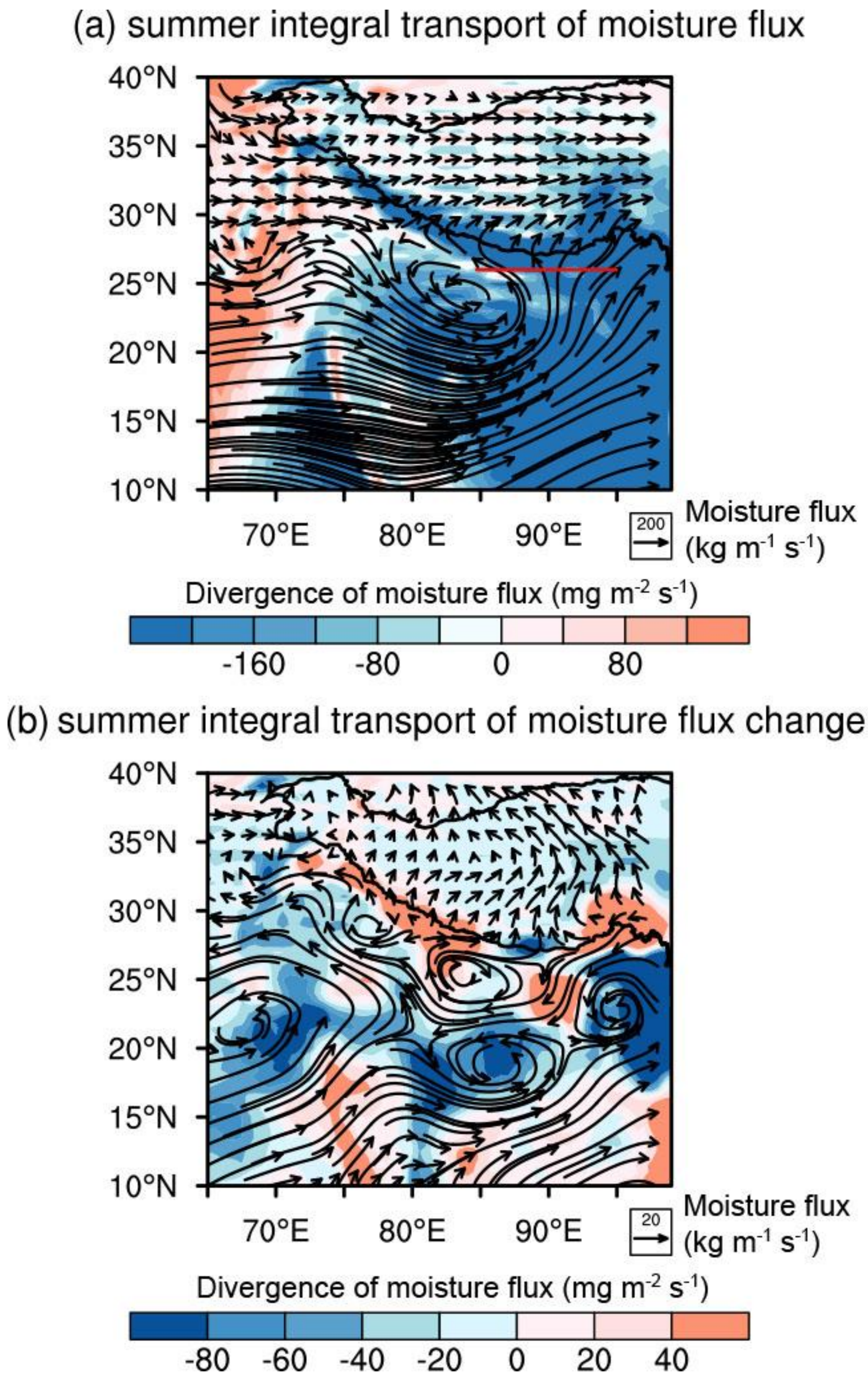

Supplementary Fig. 17 Summer integrated transport moisture flux field and its divergence derived using the ERA-Interim reanalysis dataset. (a) Summer mean during 1989 to 2018. (b) Difference between the periods 1989–2003 and 2004–2018 (the later minus the former). Red line drarn along the northern side of the Bay of Bengal is reference locus.

A previous study by Lin et al[60]. found that the summer moisture flux to the Tibetan Plateau along the northern side of the Bay of the Bengal (marked with a red line in Supplementary Fig. 17a) had reduced during the 21st century (Supplementary Fig. 18). As noted by Curio et al[61], the northern side of the Bay of the Bengal regulates the precipitation variability over the Tibetan Plateau, and this is consistent with the years of strong and weak precipitation over the plateau[60]. As observed by the 5-year smoothed red line of Supplementary Fig. 18, the year 2004 is indeed an inflexion point leading to an ensuing decrease in incoming moisture flux to the Southern Tibetan Plateau.

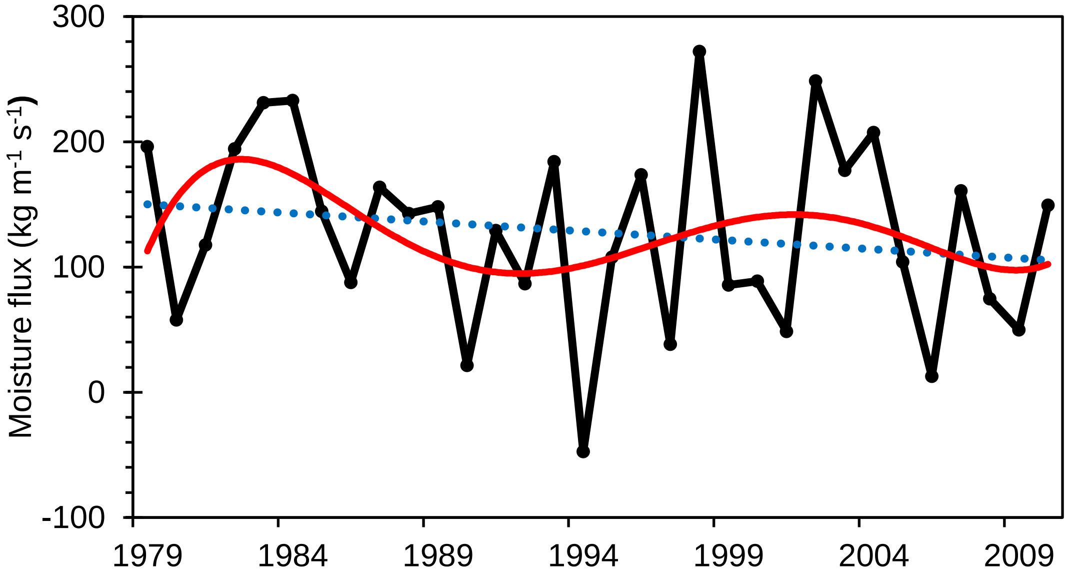

Supplementary Fig. 18 Annual change of moisture flux along the path of northern side of Bay Bengal (marked with a red line in Supplementary Fig. 17a) to the Tibetan Plateau referring to Lin et al[60]. The actual trend of moisture flux over time is delineated by the black solid line. The blue dotted line represents the fitted trend and red line is a 5-year smoothed average.

In conclusion, by analyzing the moisture flux and its divergence from various perspectives, it was discovered that an inflexion point occurred in 2004 manifested thereafter by a decreasing trend of incoming moisture flux over the southern Tibetan Plateau. Moreover, other climate dynamics also changed the most noticeable one being a strengthening of moisture flux convergence over South Asia.

3. South Asian black carbon emissions, loadings, and atmospheric heating

To investigate black carbon emissions over South Asia, we utilized monthly global black carbon emission inventories split out by sector (energy production, industry, transportation, residential and commercial, agriculture, as well as deforestation and wildfire), at 0.1°×0.1° resolution; these data were obtained from Peking University. As shown in Fig. 19, the temporal trend and variation of black carbon emissions over South Asia were essentially on an upward trend from 1961. Moreover, the difference when compared to the global average has become larger since 2001. We make note of the 2001 onwards spread vis-à-vis global/south Asia as this might induce significantly higher radiative perturbations than that implied by the globally mean estimates[62].

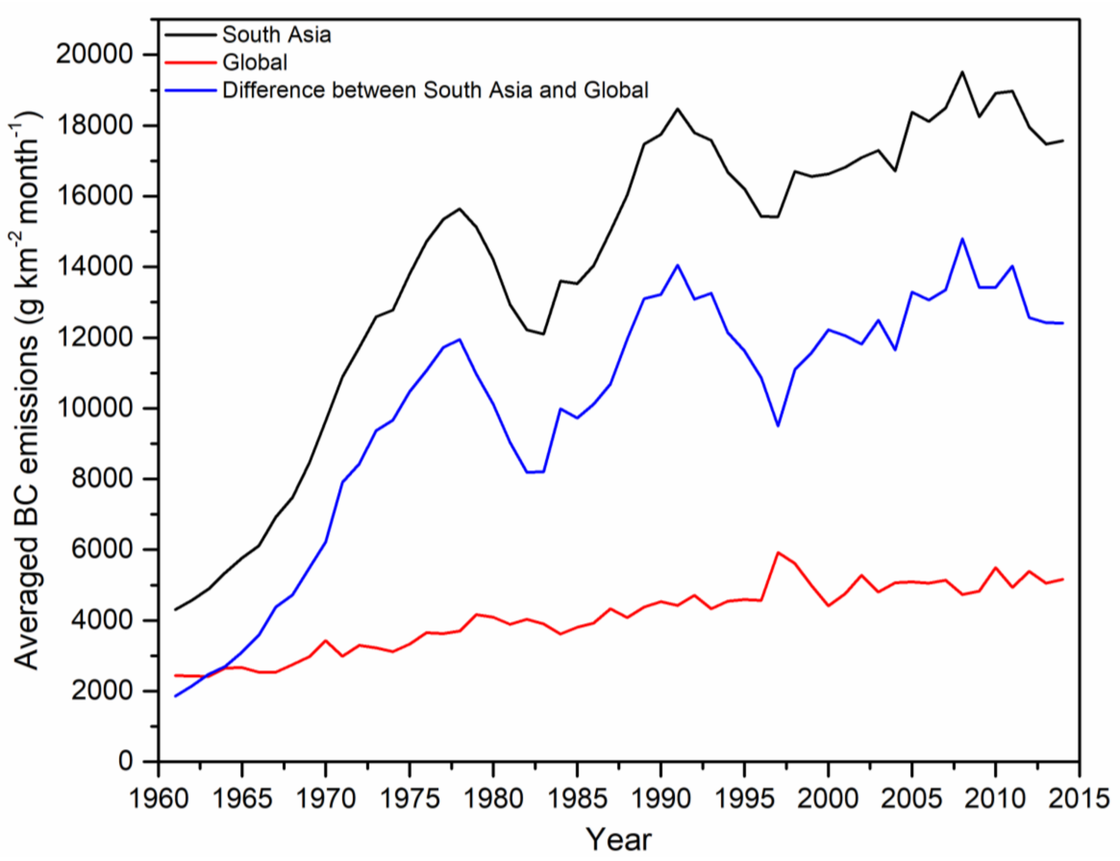

Supplementary Fig. 19 Summer monthly black carbon emissions since the 1960 over South Asia and the world from emission inventories from Peking University.

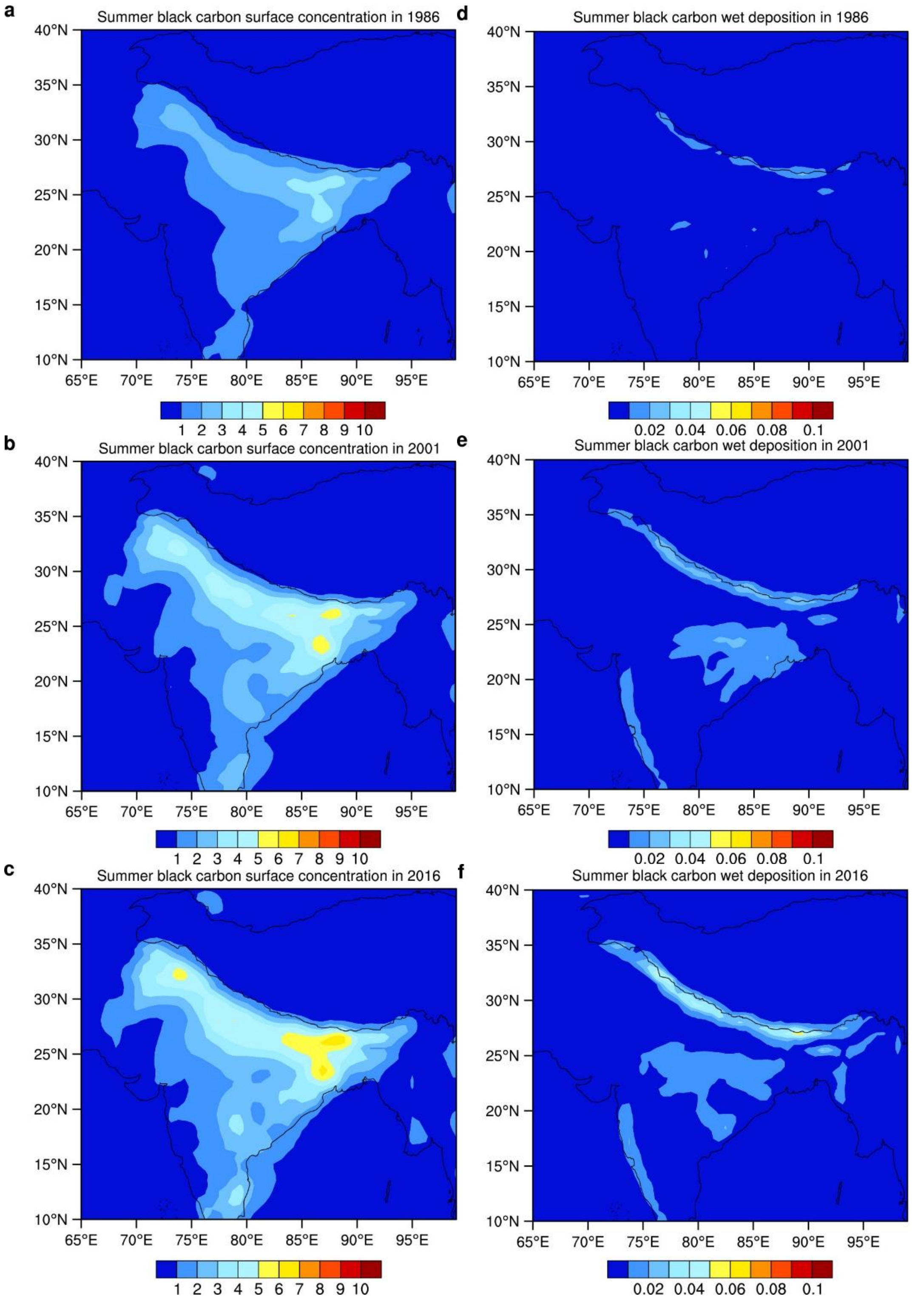

Supplementary Fig. 20 Black carbon derived from MERRA-2. Summer black carbon concentration (μg m^{-3}, **a–c**) and wet deposition (μg m^{-3}, **d–f**) over the period 1986 to 2016.

Supplementary Fig 20 is a time series of black carbon concentration and wet deposition derived from the MERRA-2 (Modern-Era Retrospective analysis for Research and Applications version 2) reanalysis dataset. An increasing trend in black

carbon emissions does not necessarily mean increasing atmospheric black carbon concentrations in the Indian subcontinent as atmospheric aerosols are largely removed during the Indian summer monsoon. To uncover the trends in atmospheric black carbon loading, the latest version of the global atmospheric MERRA-2 reanalysis dataset was analyzed. It was discovered that atmospheric black carbon concentrations have been ever-increasing with time (Supplementary Fig. 20a to Supplementary Fig. 20c). We also investigated the wet removal of black carbon during the summer monsoon, which have been also ever-increasing from 1986 to 2016 (Supplementary Fig. 20d to Supplementary Fig. 20f).

To examine the impact of South Asian black carbon on summer air temperatures, the WRF-Chem regional model was tuned and optimized to run simulations for each year from 2007 to 2016 (Supplementary Fig. 21). The results of the WRF-Chem simulations showed that increased atmospheric black carbon loading does indeed result in an increasing temperature change in the atmosphere.

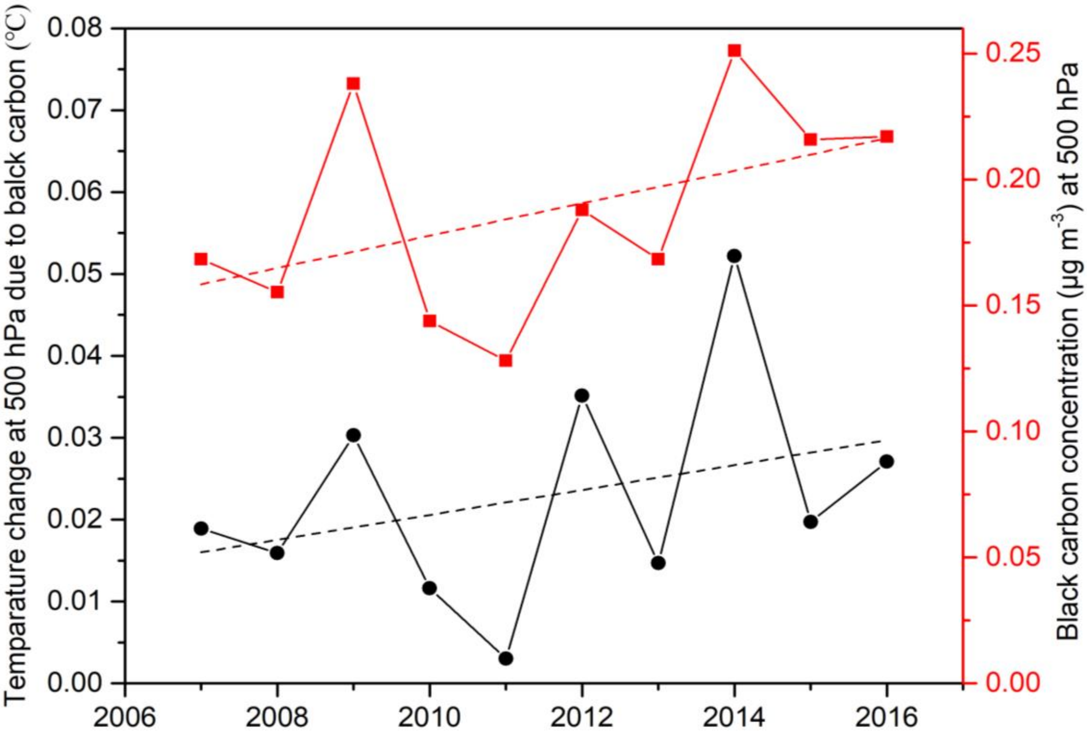

Supplementary Fig. 21 WRF-Chem simulated black carbon concentrations (red line) and subsequent warming at 500 hPa (black line) area-averaged over South Asian from 2007 to 2016. Dotted lines are fitted trend.

4. South Asian black carbon linkage with to summer precipitation decrease in south Tibetan Plateau

In the main text, we identified a decreasing trend in the summer precipitation over the southern Tibetan Plateau at an inflexion point around 2004; this decline, it was discovered, is regulated by the long-range moisture transport into the region. Further analysis was conducted to examine the change of long-range moisture transport into the southern Tibetan Plateau and, it was found that a reduction had occurred after the inflexion point. The analysis also revealed a well-defined increase in moisture convergence in South Asia, i.e., in the upstream part of the long-range moisture transport.

A review of past research conducted in South Asia[52,63] revealed that black carbon can enhance atmospheric moisture convergence conditions and increase local atmospheric convection. Given the backdrop of earlier research on black carbon we undertook a statistical analysis whereupon results showed that the summer precipitation regime over the south Tibetan Plateau from 2004 onwards had a negative correlation with the south Asian summer black carbon emissions. A contrast, however, was found over the entire timeline of the dataset i.e., a positive correlation. Finally, we undertook a WRF-Chem model investigation of potential mechanism(s) that might be associated with South Asian black carbon and moisture transport change and, subsequent observed alteration of the summer precipitation over the southern Tibetan Plateau.

4.1 The lag correlation analysis between black carbon and summer precipitation

As was plotted in Fig. 1 in the revised manuscript, black carbon emissions have been increasing since 1985 but the summer precipitation observed decrease in the southern Tibetan Plateau occurred later at the inflexion point of 2004. Given the aforementioned, one would postulate there exists a delay / tipping point between the two. To establish as to whether a delay / tipping point exists between the two, we conducted a lag correlation analysis between South Asian black carbon and summer precipitation over the southern Tibetan Plateau. As can be seen in Supplementary Fig.

22, there is an insignificant ($P > 0.05$) lag relationship between summer precipitation over the Tibetan Plateau and South Asian black carbon; this is especially so for a lag time of more than 10 years. Thus, we concluded that the negative correlation is more than likely due to black carbon reaching a critical level in the atmosphere to affect local convection and precipitation. Returning to Supplementary Fig. 19 once again, i.e., the difference in South Asian black carbon emission compared to the global average, we remarked upon the larger divergence between the two since the 21th century. We also referenced Ramanathan et al[62]. to infer that the divergence may well induce significantly higher radiative perturbations than the globally mean estimates.

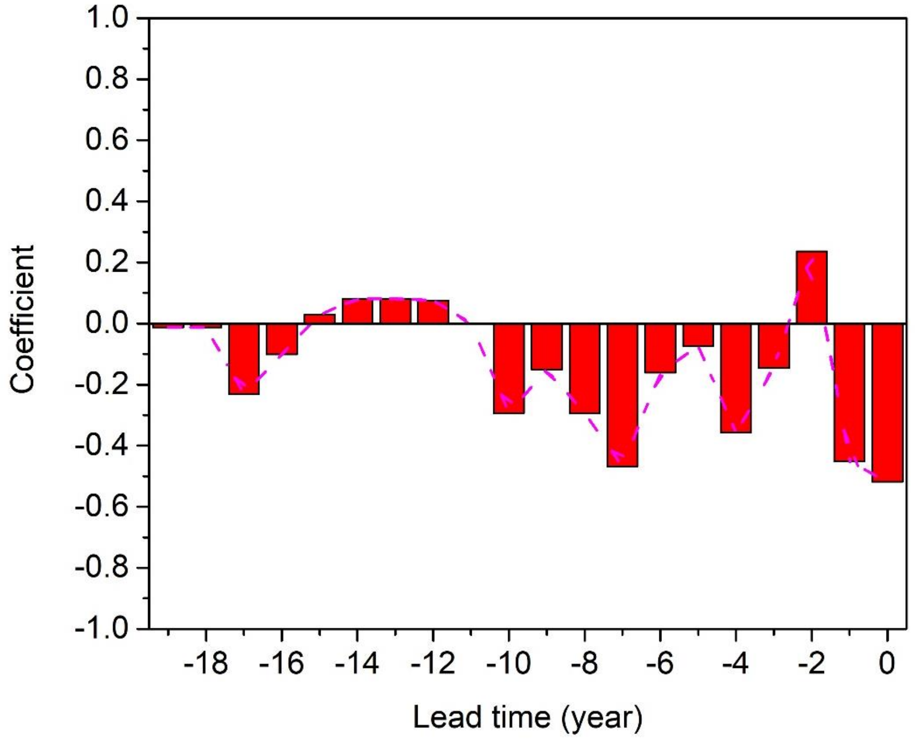

Supplementary Fig. 22 Cross correlation between summer area-averaged precipitation in the southern Tibetan Plateau and South Asian area-averaged black carbon emissions starting at 2004 lagged out to 19 years of black carbon emissions. Dashed line represents smoothed fit.

As a further test of the WRF-Chem capacity to simulate actual conditions we reversed the hypothesis, i.e., can the model not model a condition. Hence, we conducted WRF-Chem simulation experiments for 2016 using the South Asian black

carbon inventory for 1985 where black carbon emissions were at a lower level; this was to explore whether lower concentrations would drive the same climate dynamics as was found to be the case for the 2007–2016 period (Supplementary Fig. 4a, b in the revised manuscript). The model simulations were unable to reproduce a strengthening of the cyclonic circulation of moisture in South Asia during the summer (Supplementary Fig. 23a, cf. Supplementary Fig. 4a). In addition, the northward transport of moisture flux from South Asia to the southern Tibetan plateau was insignificantly weakened. The moisture flux along 80°E (Supplementary Fig. 23b) indicates that 1985 forced black carbon was not an adequate amount to result in a water vapor increase over South Asia with a concomitant reduction over the southern Tibetan Plateau. In essence then, summer precipitation over the southern Tibetan Plateau starts to decrease around 20 years after 1985 and is not a lagged effect.

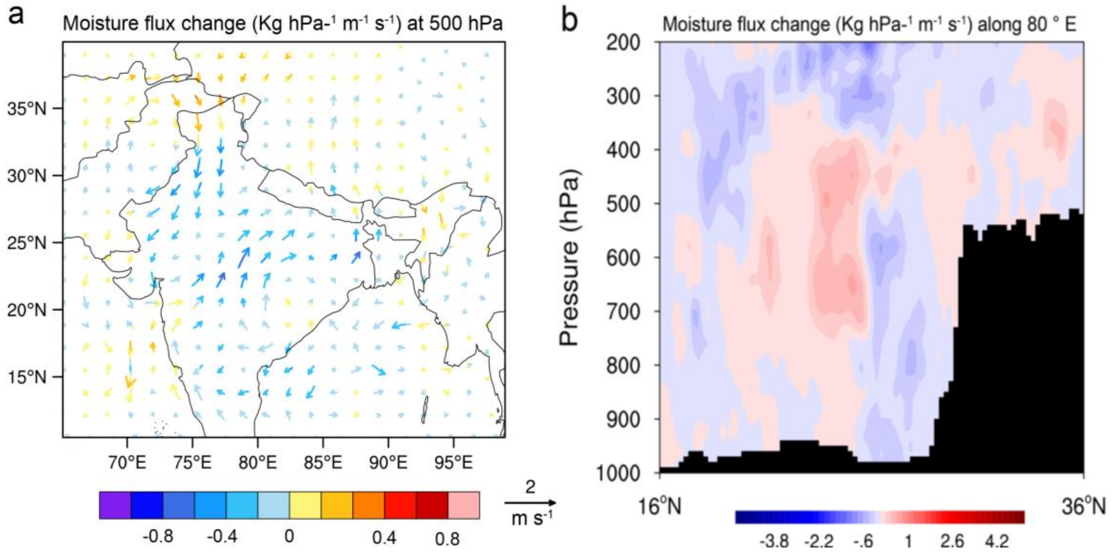

Supplementary Fig. 23 South Asian black carbon triggered summer moisture flux change in 2016. a at 500 hPa and **b** along 80 °E using the1985 emission inventory.

In summary, through combined lag-time correlation analysis and WRF-Chem model simulations, it appears that there is no delayed effect of increased South Asian black carbon on reduced summer precipitation over the southern Tibetan Plateau. The change in meteorological fields (meridional moisture and vertical wind components) since 2004 are attributed to the contemporaneous South Asian black carbon emissions reaching a sufficient level that significantly affects local convection and precipitation.

4.2 Mechanism of South Asian black carbon affecting on precipitation

Using the WRF-Chem model, a series of sensitivity simulations were undertaken to examine the climate dynamics and forcing due to atmospheric black carbon over south Asia. A dominant summer moisture flux change identified was an intensification of the cyclonic circulation of moisture in the eastern Indian subcontinent and the Bay of Bengal in the lower-layer atmosphere (Supplementary Fig. 24). As to the summer precipitation decline that has occurred over the southern aspect of the Tibetan plateau; this is discussed in the revised text. In addition to the direct radiative effect, black carbon can also influence the properties of liquid clouds and ice clouds. It has been shown that cloud condensation nuclei (CCN) can increase with increasing aerosol loading[64,65,66] (Fan et al., 2013; Rosenfeld et al., 2008; Yuan et al., 2008). The past documented research is very relevant given that Supplementary Fig. 25 highlights the increased summer CCN number concentrations at 1% supersaturation in the troposphere over the Indian subcontinent.

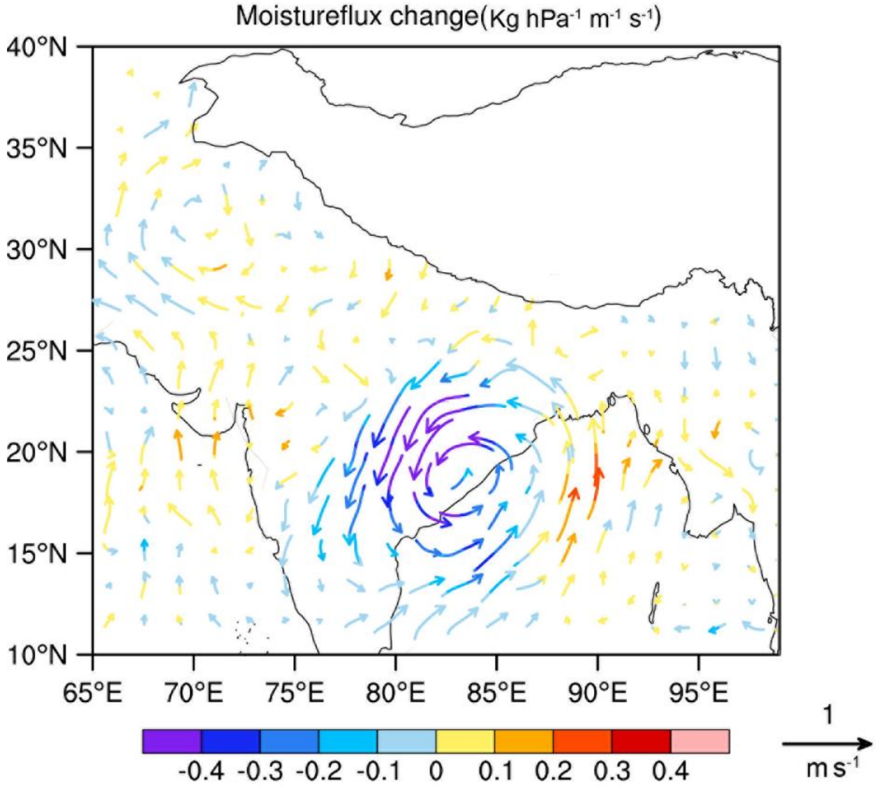

Supplementary Fig. 24 850 hPa WRF-Chem simulated average summer moisture flux change due to South Asian black carbon loading for the period 2007–2016.

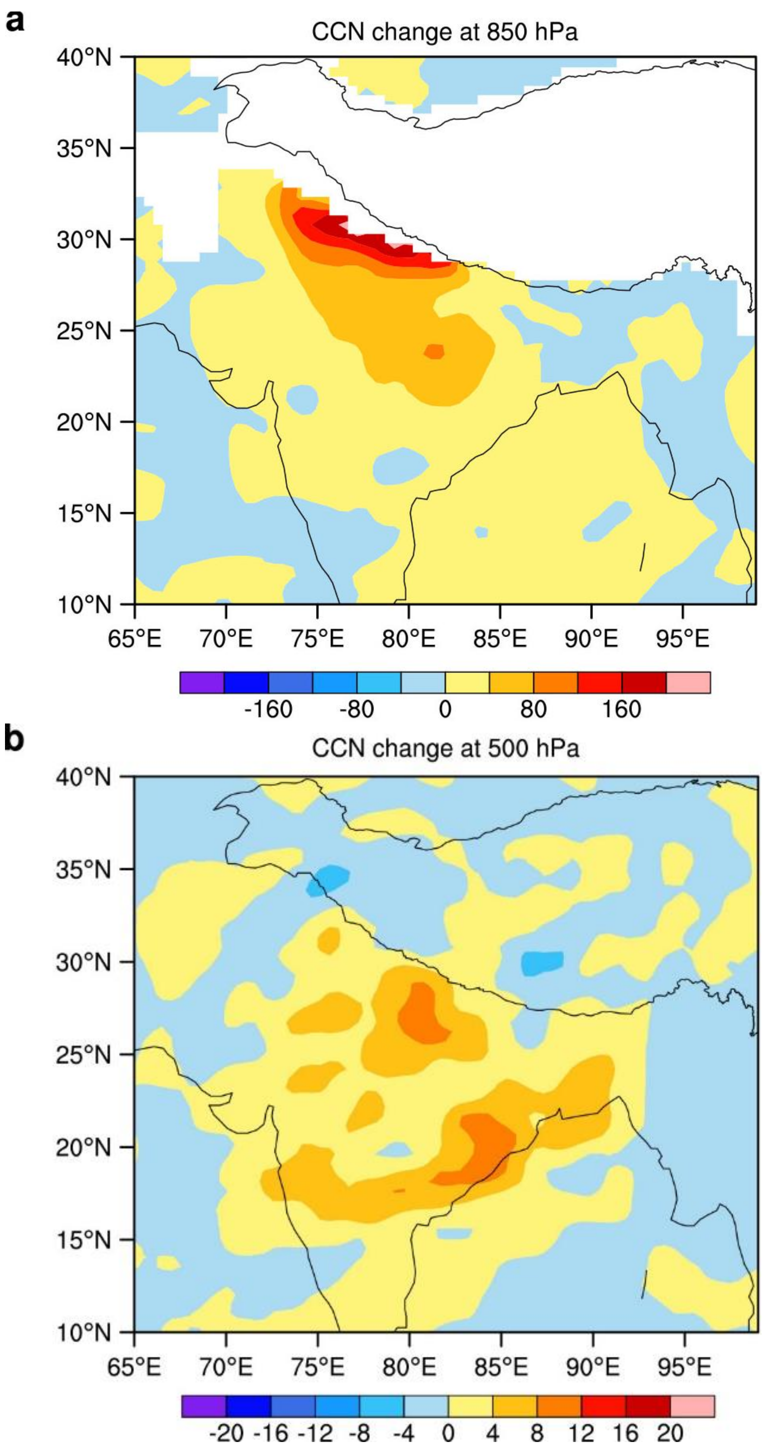

Supplementary Fig. 25 Summer average CCN (cm^{-3}) change at 1% supersaturation due to South Asian black carbon loading for the period 2007–2016. a 850 hPa and **b** 500 hPa.

Another central factor that our analysis uncovered is that of convective available potential energy (CAPE). Supplementary Fig. 4c in the main text, confirms that there has been a significant increase in CAPE in the eastern Indian subcontinent. Intensified black carbon concentrations which is an absorbing aerosol, induces positive radiative

forcing and subsequent heating of the low and middle troposphere (Supplementary Fig. 26b). Moreover, air temperature and change (as plotted in Supplementary Fig. 26) reflects the fact that black carbon induces increased meridional temperature gradients in the eastern Indian subcontinent, i.e., northern-increased versus southern-decreased.

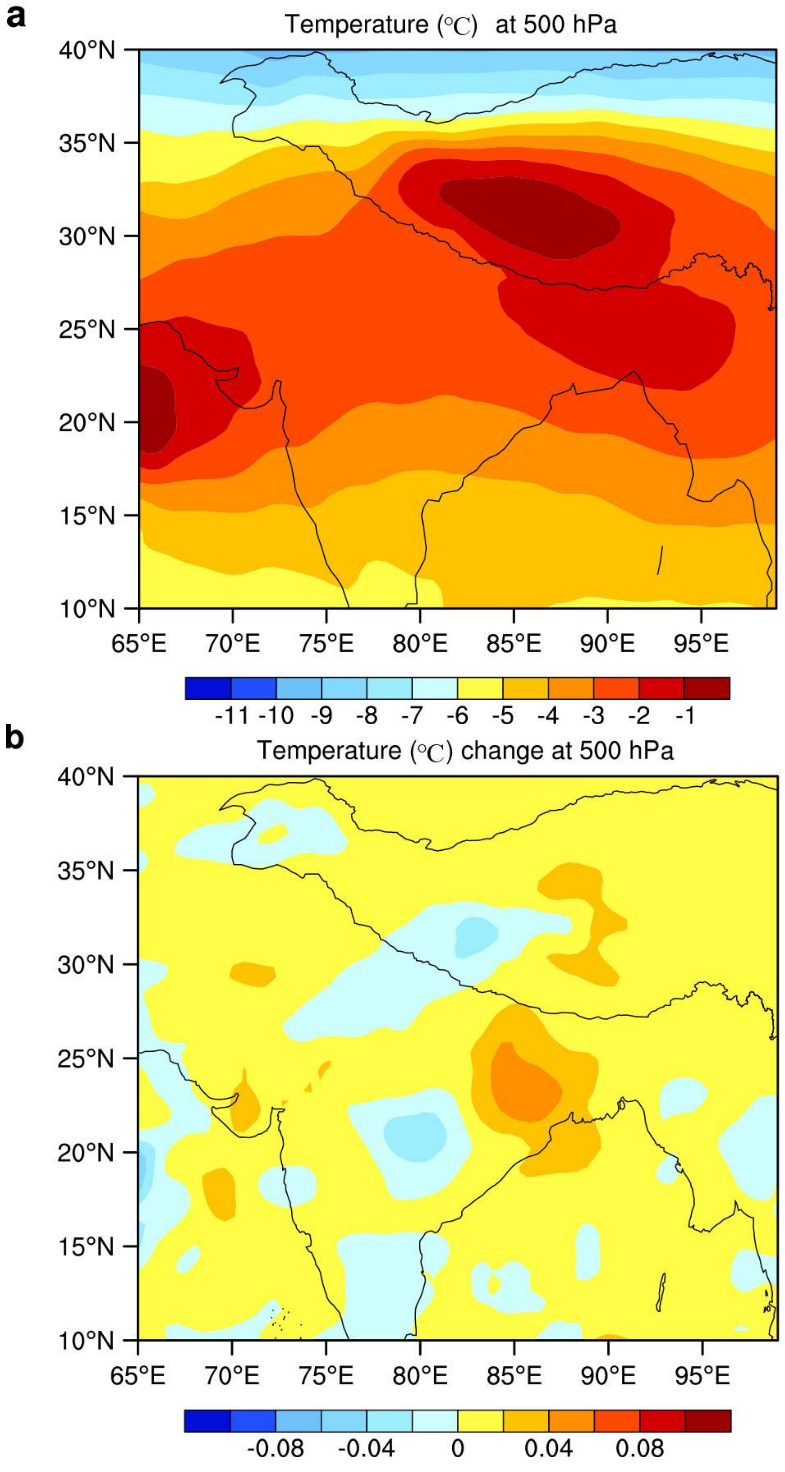

Supplementary Fig. 26 500 hPa WRF-Chem simulated temperature for the period 2007–2016. **a** summer mean temperature and **b** its change caused by South Asian black carbon loading.

The enhancement of local convection and moisture source over the Indian subcontinent resulted in higher summer cloud water mixing ratios over that region (Supplementary Fig. 27). As a consequence, localized increased precipitation reduced water vapor availability elsewhere and, as noted earlier, was coupled with a weakening downstream flow in the direction of the southern Tibetan plateau; the change in the induced regional dynamics ends up as reduced precipitation.

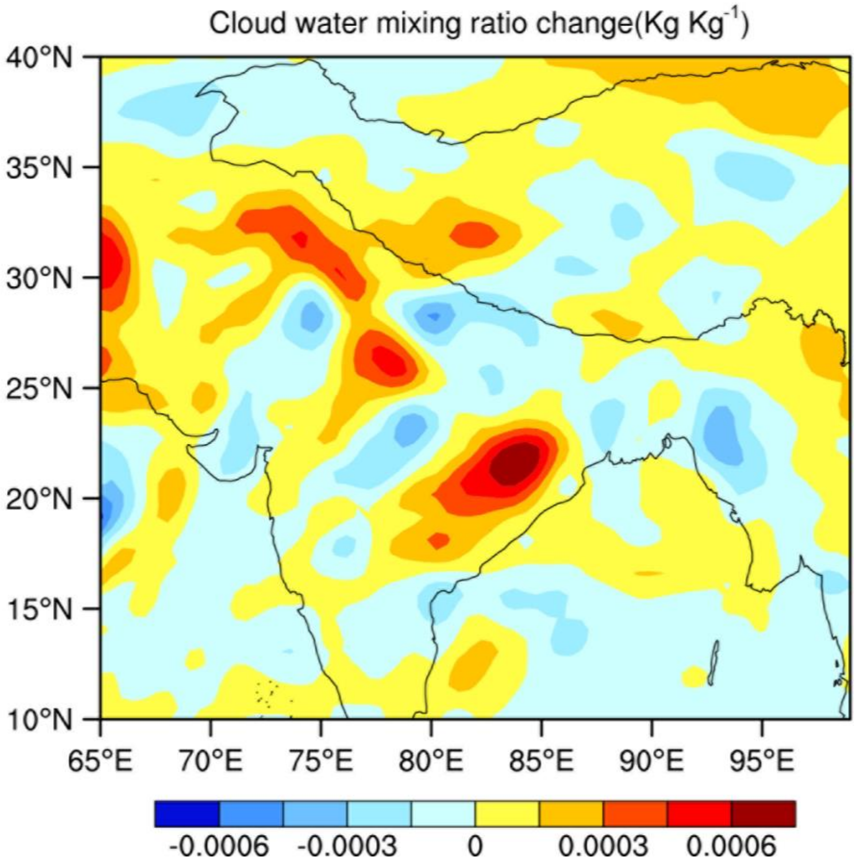

Supplementary Fig. 27 WRF-Chem simulated cloud water mixing ratio change caused by South Asian black carbon induced summer mean cloud water mixing ratio change during 2007–2016.

Additional analysis was carried out for severe precipitation events over the Indian subcontinent: 228 heavy rain days were selected from the APHRODITE daily gridded precipitation dataset: these accounted for 39.6% of total summer precipitation during 2007–2015. A threshold value of that exceeding 100 mm/day defined a heavy rain day. The results (Supplementary Fig. 28) of area-averaged daily rainfall show unsurprisingly that daily heavy rain averaged over South Asia was accompanied alongside comparatively lower values for the southern Tibetan plateau.

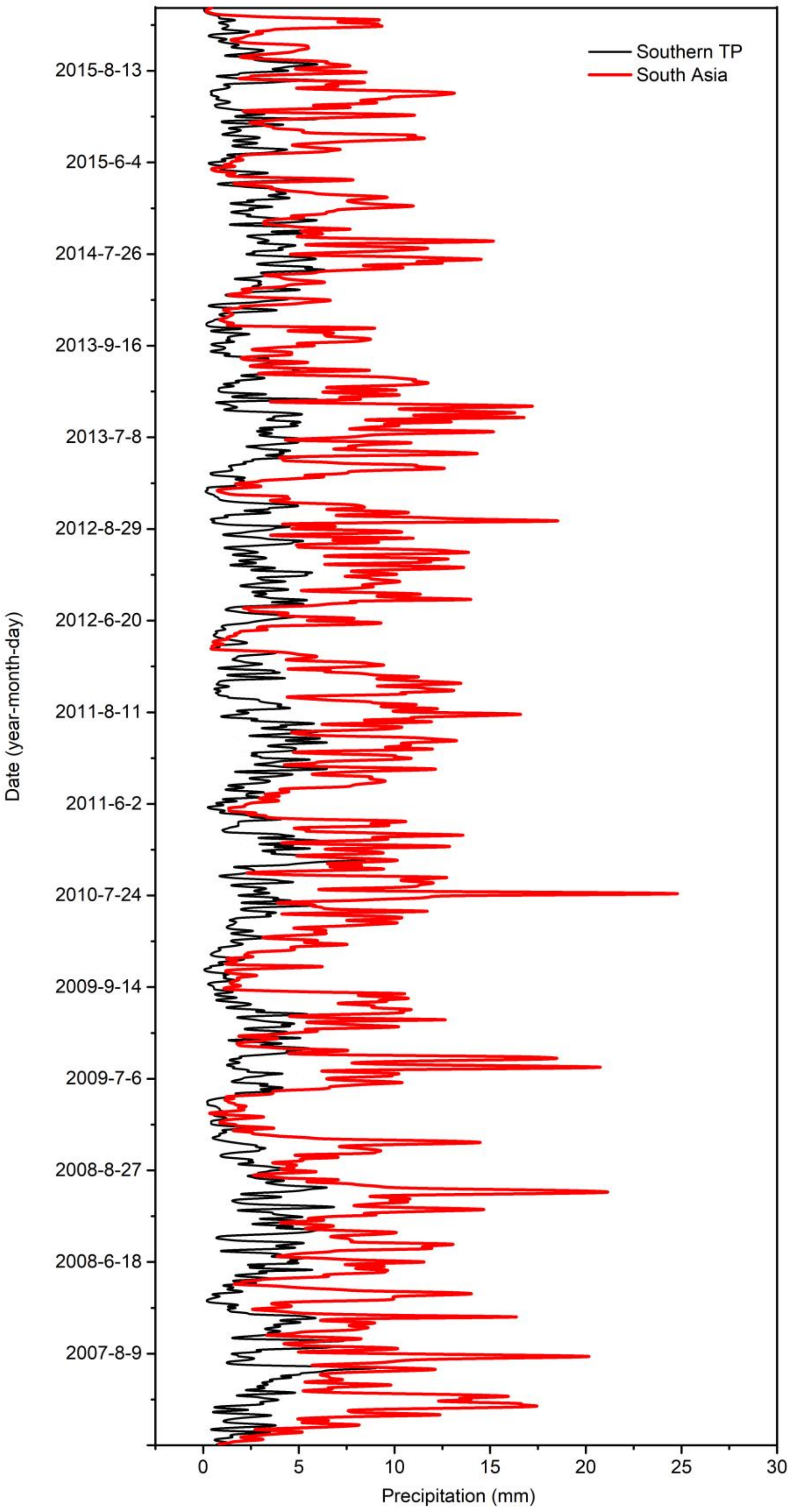

Supplementary Fig. 28 Comparison of summer area-averaged daily rainfall (APHRODITE daily dataset) over the southern Tibetan plateau with that over South Asia for the period 2007–2015.

As discussed earlier, the WRF-Chem model was turned and optimized to study the effects of increasing black carbon. With respect to the precipitation event part i.e., heavy rain days, simulations indicated that black carbon lead to increased daily precipitation (Supplementary Fig. 29a) for most of the Indian subcontinent and, the results were consistent with the APHRODITE maximum precipitation centers (black dots in Supplementary Fig. 29a). Moreover, the WRF-Chem breakdown into the two components of convective and large-scale precipitation revealed (a) enhancement of the convective precipitation regime over the Indian subcontinent (Supplementary Fig. 29b), while at the same time (b) a decrease in large-scale precipitation (Supplementary Fig. 29c). The results reinforce prior lines of inquiry as to the effects of black carbon and enhanced convective instability, increased summer heavy rain and depletion of atmospheric water that is subsequently transported northward to the southern Tibetan plateau.

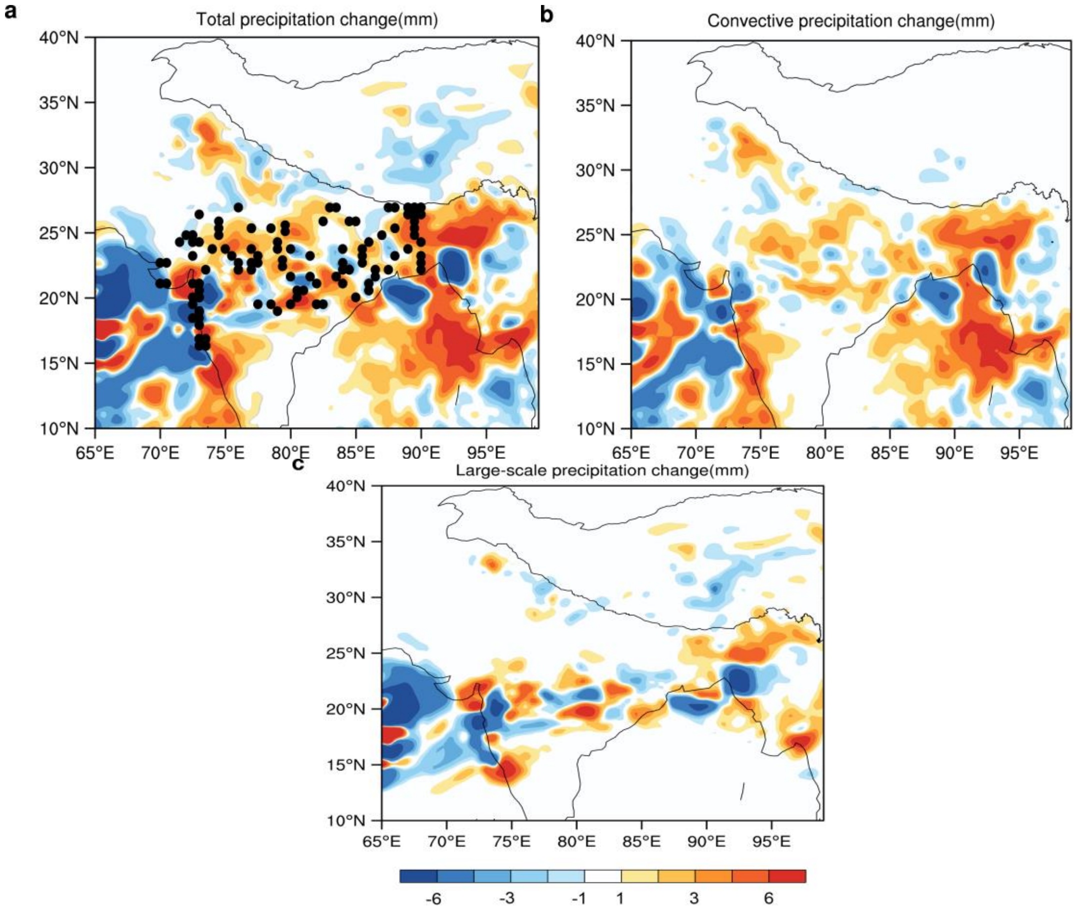

Supplementary Fig. 29 South Asian black carbon triggered average change for 228 heavy rain day events for the period 2007–2016. a total precipitation, **b** convective precipitation, and **c** large-scale precipitationBlack dots in **a** represent the APHRODITE maximum precipitation centers.

5. Glacial mass balance change and its climate drivers

5.1 Glacial mass balance change over the Tibetan Plateau

The Tibetan Plateau and surrounding areas contain the largest number of glaciers outside of the polar regions. According to Maurer et al., 2019, the Himalayan glaciers have experienced the most intensive ice loss over the past 40 years. Tibetan Plateau glacial shrinkage generally decreases from the Himalayas to the continental interior and is the least in the eastern Pamir with positive mass balance[67].

To investigate the extent of glacial mass decline, we adopted the method proposed by Brun[68], i.e., time series of digital elevation models (DEMs) were used to calculate the glacier volume changes for the 2007–2016 period. The methodology is fully automated and calculates DEMs from available ASTER satellite optical stereo pairs. A linear regression through time series of co-registered ASTER DEMs was fitted to estimate the rate of elevation change for each 30-m pixel. A particular strength of this method is that it relies exclusively on satellite optical data. Thus, it is not affected by signal penetration, which is a major source of uncertainty in DEMs derived from radar sensors (e.g., from the Shuttle Radar Topography Mission; SRTM), in which the signal penetrates to an unknown depth up to many meters into the snow and ice[69]. Supplementary Fig. 30 shows the spatially distributed differences in glacier mass balance. The most prominent positive mass change (up to 0.3m water-equivalent-year^{-1}) is observed at West Kunlun, in the northwestern part of the Tibetan Plateau. The largest glacial mass loss was found in Nyainqentanglha with a variability of −0.53m water-equivalent-year^{-1} in Nyainqêntanglha.

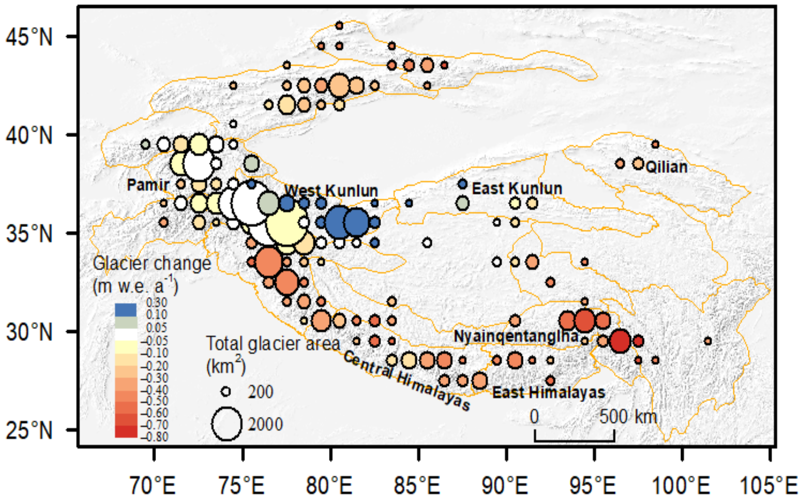

Supplementary Fig. 30 The summer average glacier volume change over the Tibetan plateau from 2007 to 2016. The base map image is generated by the first author, based the downloaded Digital Elevation Model (DEM) data from the open source website (http://www.gscloud.cn/sources/?cdataid=302&pdataid=10).

In addition to the glacier volume calculation, we reconstructed the time series of reference-surface mass balance for the 1979–2014 period using an empirical model (more details in section 1.4) calibrated with observed mass balance from 45 glaciers. Supplementary Fig. 31 shows the time series evolution of cumulative annual glacier mass balance. There is insignificant variability before year 2000 (−0.57m water-equivalent-year^{-1}). However, there is accelerated glacial mass loss after year 2000 with cumulative glacier mass balance of −2.97m water-equivalent-year^{-1}. Striking through is the glacial mass decline in the central Himalayas (−13.54m water-equivalent-year^{-1}) and the Nyainqentanglha (−24.38m water-equivalent-year^{-1}). Moreover, glaciers in the central Himalayas and the Nyainqêntanglha have experienced greater cumulative annual glacial mass balance loss when compared to the mean. One inference that may be taken from the glacial loss differences may be that the southern Tibetan Plateau is more sensitive to climate perturbations that are anthropogenic in nature.

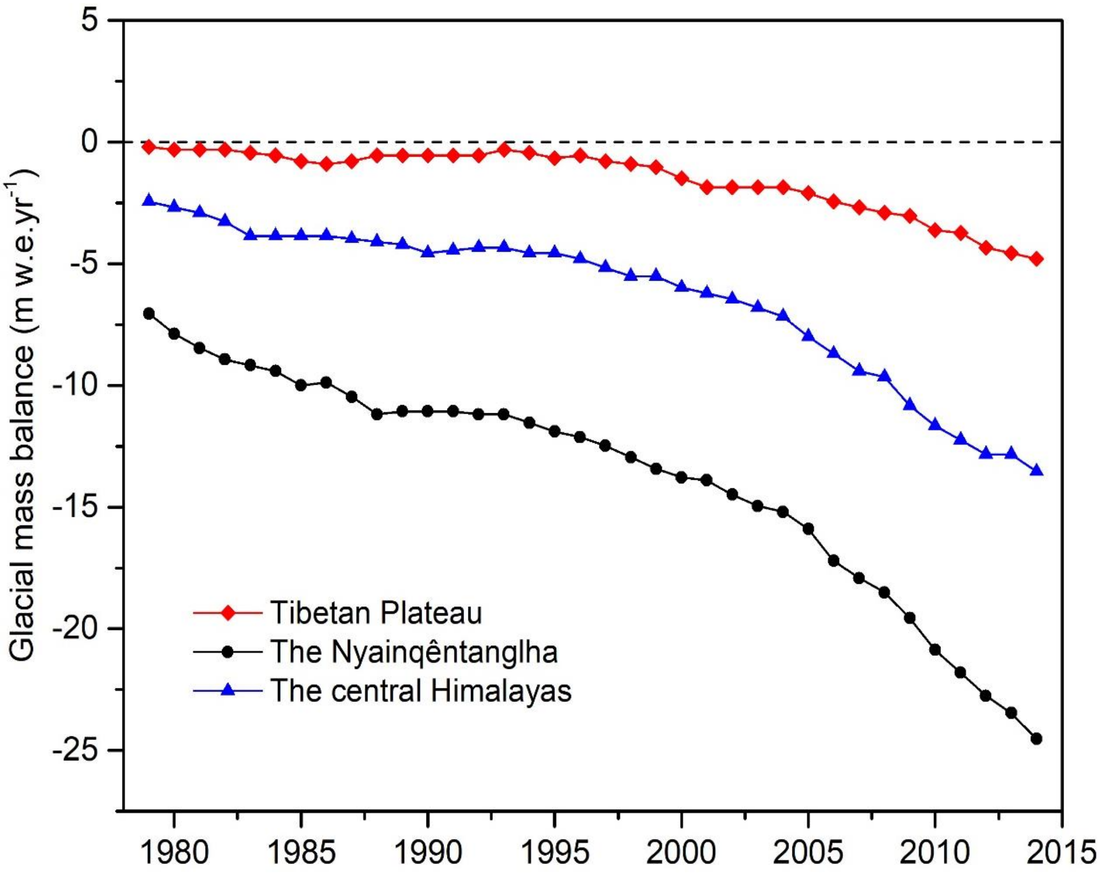

Supplementary Fig. 31 The cumulative annual glacier mass balance over the Tibetan Plateau as well as in the central Himalayas and, the Nyainqêntanglha over the southern Tibetan Plateau.

5.2 Drivers of glacial mass balance change

In order to determine the drivers of glacial change, we applied the optimization procedure from Radić and Hock[57] in the calibration of an empirical model (more details in section 1.4) using observed mass balance time series data from 45 glaciers over the plateau[70] and obtained the best match. The empirical model was used to investigate the mass balance sensitivities of each 45 glaciers to temperature changes from −6 to 6 Kelvin and precipitation changes from −30% to 30%. The results (Supplementary Fig. 32) show a difference in sensitivity to temperature and precipitation: (i) the change in mass balance linearly increases with the increase in precipitation perturbation but (ii) the relationship between the change in glacial mass balance and the change in temperature perturbations is non-linear.

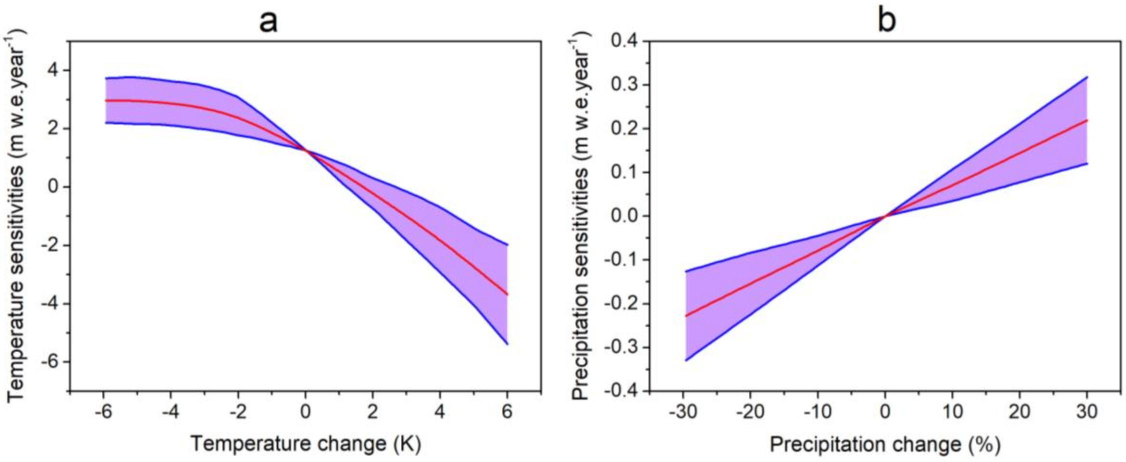

Supplementary Fig. 32 Glacial mass balance sensitivities to climate drivers following Wang et al[70]. a temperature and **b** precipitaion. The red line indicates the mean sensitivity across the 45 glaciers, and the violet shading reflects the standard deviation.

Applications of the empirical model also uncovered significant spatial heterogeneity in the annual glacial mass balance for 1979 to 2014 period; this is shown in Supplementary Fig. 33(a). The patterns of glacial mass gain in Kunlun and southern Tibetan Plateau mass loss (e.g., the Himalayas and Hengduan Mountains) are in agreement with the results obtained from other distinctive studies, i.e., ICEsat[71], ASTER[68], and GRACE[72]. Although rising temperatures are considered the dominant driver of glacier change in the Himalayas [73,74], changes in precipitation in both directions likely drive the systematic differences in glacier status over the Tibetan Plateau[67]. Besides, it is worth reiterating that summer precipitation accounts for more than 60% of the total annual precipitation that feeds the Tibetan plateau[75,76]; this rises to 90% over the southern Tibetan Plateau.

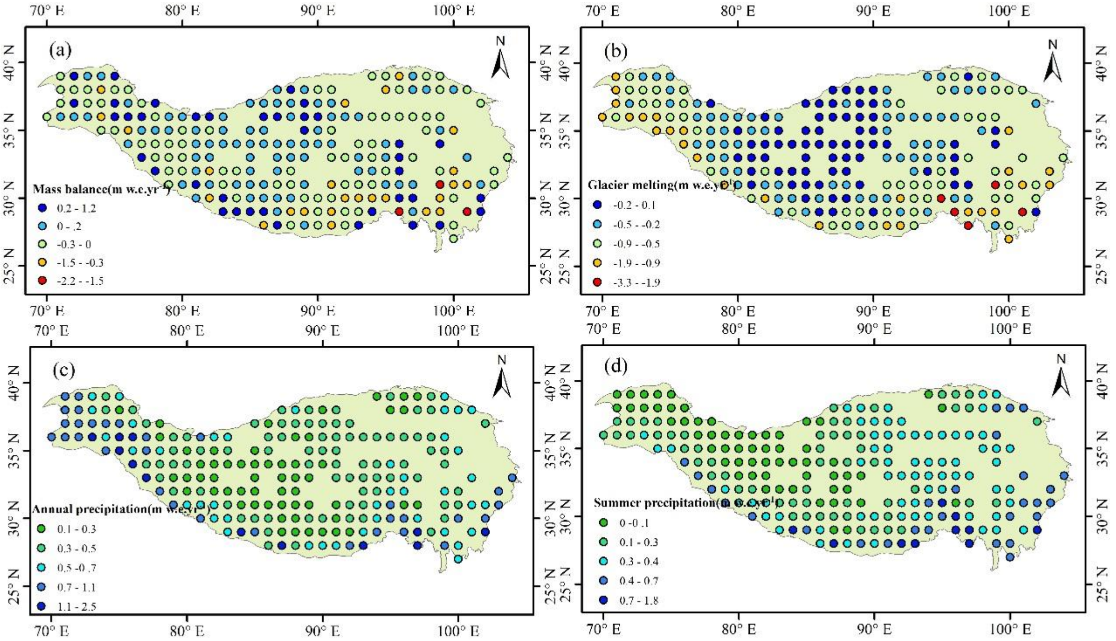

Supplementary Fig. 33 Spatial patterns of glacial mass balance as well as its changes due to different climate drivers simulated by the glacier mass balance model. a annual mean glacial mass balance and its changes due to **b** temperature, **c** annual precipitation, and **d** summer precipitation for the period 1979–2014.

Supplementary Fig. 33 also shows the individual contribution of precipitation and warming to the annual mean glacial mass accumulation: the glacier mass change of glaciers over the Tibetan Plateau is driven by both temperature (Supplementary Fig. 33b) and precipitation (Supplementary Fig. 33c). Moreover, precipitation has resulted in substantive change in glacial mass over the southern Tibetan Plateau, especially in the Himalayas. As reported by a plethora of previous analyses, summer precipitation affects the glacial mass balance in the southern Tibetan Plateau more than that in the northern parts (Supplementary Fig. 33d).

Supplementary Fig. 34a shows the analysis of time series in glacial mass balance and its climate drivers over the Tibetan Plateau. It would seem that glacial melting by temperature is the dominant driver of glacier mass loss. Meanwhile, over time precipitations' contribution to glacial mass accumulation is unchanged. However, things are somewhat different for precipitation in the central Himalayas (southern Tibetan Plateau) as Supplementary Fig. 34b indicates a declining trend with greater variability. Moreover, the contribution from summer precipitation accounts for a larger proportion of the contribution from the annual precipitation.

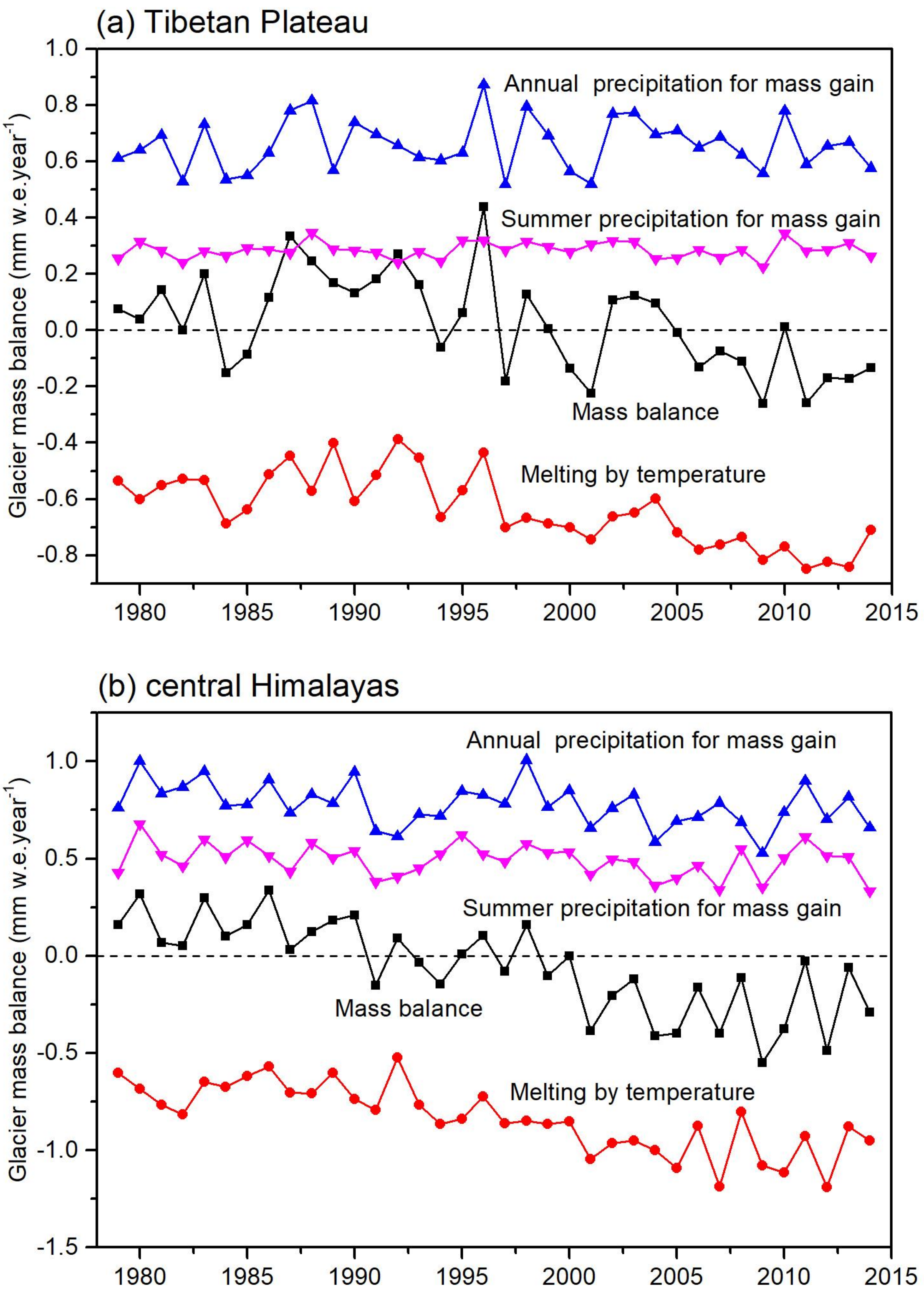

Supplementary Fig. 34 Time series of glacial mass balance as well as its changes due to different climate drivers. **a** the Tibetan Plateau and **b** the central Himalayas, southern Tibetan Plateau.

Finally, considering the important role of precipitation in glacial mass balance over the southern Tibetan Plateau, we investigated whether there has been a seasonal and decadal shift in precipitation, as well as its contribution to glacier mass accumulation. To account for shifts in precipitation over the southern Tibetan Plateau, we conducted a PDF (probability density function) analysis and found that the precipitation exhibited a marked seasonal pattern with significantly greater precipitation in the summer (Supplementary Fig. 35a). The contribution of summer precipitation to glacial mass accumulation (Supplementary Fig. 33d) actually accounts for a large proportion of the contribution from the annual precipitation (Supplementary Fig. 33c). Also, there is a decadal shift in precipitation over the southern Tibetan Plateau (Supplementary Fig. 35b), forcing a decadal shift of summer precipitation to contribute to glacier mass accumulation (Supplementary Fig. 35c).

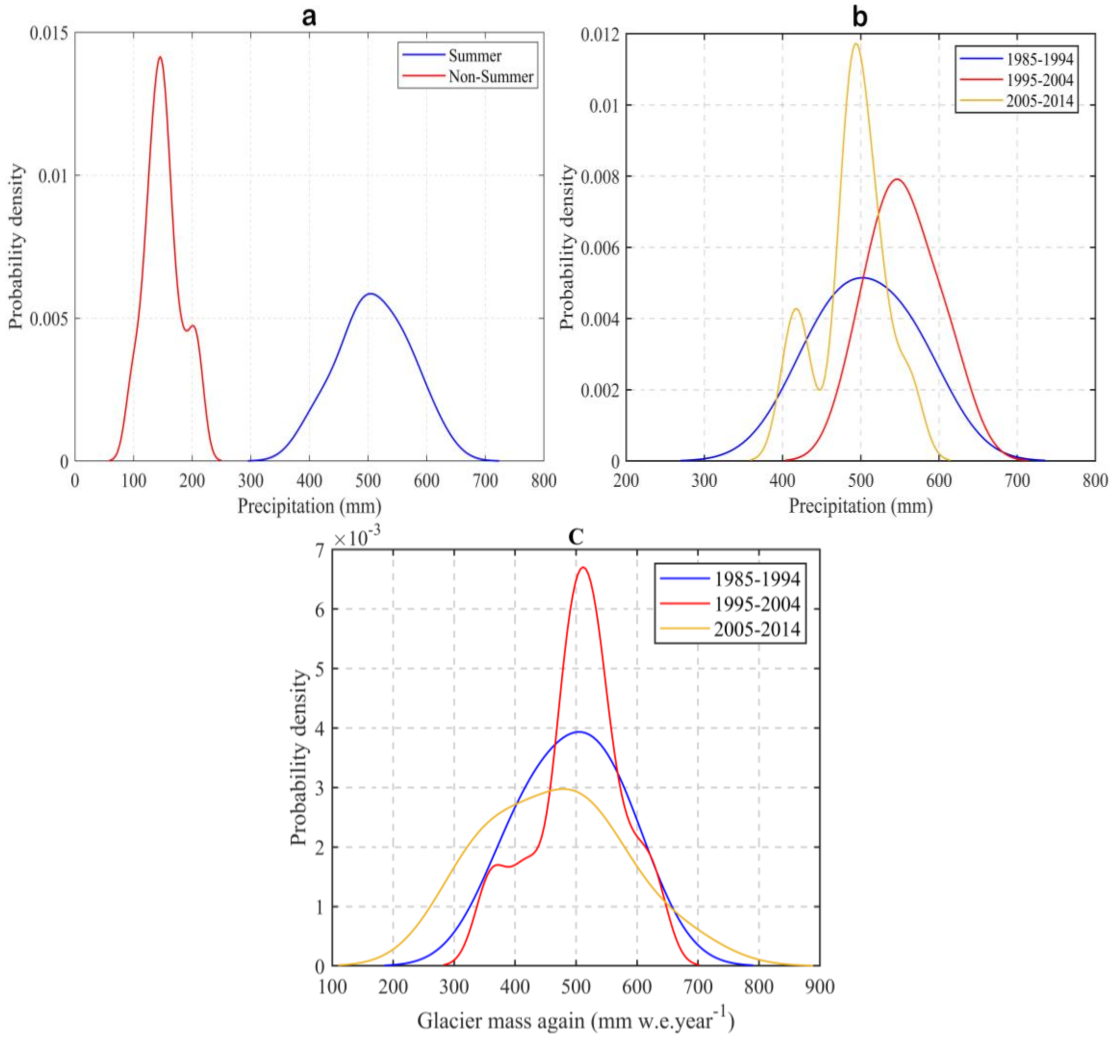

Supplementary Fig. 35 The probability density functions. a seasonal precipitation during 1979–2014, **b** decadal summer precipitation and **c** their contribution to glacial mass accumulation for the 1979–2014 period.

6. Supplementary information for datasets used in this work

Here, we supplemented more details for the datasets used in this study, as shown in Supplementary Table 7, including the data type, spatial and temporal resolutions, and the time/period used.

Supplementary Table 7 More details of the datasets used in this study

	Dataset name	Type	Resolutions (spatial, temporal)	Period
Precipitation	CRU	Gridded	0.5°×0.5°, month	1961–2016
	APHRODITE	Gridded	0.25°×0.25°, day	2004–2015
	ERA5	Gridded	0.25°×0.25°,	2016
	GPCP	Gridded	2.5°×2.5°, month	1979–2016
	CMDSC	In-*situ*	China, day	2004–2016
	UCC	In-*situ*	South Asia, day	2016
Specific moisture	ERA-Interim	Gridded	0.25°×0.25°, month	1989–2018
Wind	ERA-Interim	Gridded	0.25°×0.25°, month	1989–2018
Moisture flux	ERA-Interim	Gridded	0.25°×0.25°, month	1989–2018
Divergence of moisture flux	ERA-Interim	Gridded	0.25°×0.25°, month	1989–2018
Black carbon concentration and wet deposition	MERRA-2	Gridded	0.5°×0.625°, month	1986, 2001, 2016
Black carbon concentration	APCC	In-*situ*	South Asia and TP, day	2016
Black carbon emission	Peking University	Gridded	0.1°×0.1°, month	1961–2014

*UCC data is derived from the Utah Climate Center (https://climate.usu.edu/). CMDSC data is accessible at http://data.cma.cn/.

In addition, it is a fact that different years/time periods were used which pertain to the analysis at hand. In Supplementary Table 8, we have itemized the datasets selected periods, the analysis undertaken and, a brief explanation.

Supplementary Table 8 Summary of iterative analyses and corresponding dataset periods used in this study.

Different analyses	Datasets	Note
I: Identification of a decreasing trend in summer precipitation in the STP since 2004.	CRU (1961–2016, Fig. 1b)	
	GPCP (1979–2016, Fig. S15)	This dataset is available since 1979
II: Confirmation of decreased summer precipitation in STP since 2004.	In *situ* observations (2004–2016, Fig. S16a)	
	APHRODITE (2004–2015, Fig. 1c)	This dataset is only updated to 2015
III: Comparing the change of incoming moisture to the TP before and after 2004, indicating a decrease in incoming moisture since 2004.	ERA-Interim (1989–2018, Fig.2a, Fig. 2b, and Fig. S17)	To reveal the average annual change for fifteen years before and after 2004.
IV: Discussion of the correlation coefficient between summer precipitation decreased over the STP and BC emissions over the South Asia since 2004.	Peking BC emission (1961–2014, Fig. 2c and Fig. 2d)	This dataset period is only covered from 1960 to 2014.
	CRU precipitation (1961–2014, Fig. 2c and Fig. 2d)	
V: Revealing the annual averaged effect of Asian BC on summer precipitation decrease over the STP, and its contribution to glacial mass loss.	WRF-Chem simulations (2007–2016, Fig. 3 and Fig.4)	Prior similar simulations using the MOZART represented well the BC after 2006, so 2007–2016 selected.
	InVEST evaluation (2007–2016, Fig. 5)	

*BC and STP are the abbreviations of black carbon and southern Tibetan Plateau, respectively.

7. Supplementary References

1. Wang, S. J., Yan, Y. X., Yan, M., Zhao, X. K. Quantitative estimation of the impact of precipitation and human activities on runoff change of the Huangfuchuan River Basin. *J. Geogr. Sci*. **5**, 140–152 (2012).
2. Athukorala, R., Thol, T., Neluwala, P., Petri, M., Sengxeu, S., Lattada, L., ... & Sithivong, V. Evaluating the Performance of a WRF Physics Ensemble in Simulating Rainfall over Lao PDR during Wet and Dry Seasons. *Adv. Meteorol*. **4**, (2021).
3. Jankov, I., Gallus, W. A., Segal, M., Shaw, B., & Koch, S. E. The impact of different WRF model physical parameterizations and their interactions on warm season MCS rainfall. *Weather. Forecast*. **20**, 1048–1060 (2005).
4. Skamarock, W. C., Klemp, J., Dudhia. J., Gill, D., Barker, D., Wang, W., Powers. J. A description of the Advanced Research WRF version 2. NCAR Tech. Note NCAR/TN-4681STR. (2005).
5. Grell, G. A., Peckham, S. E., Schmitz, R., McKeen, S. A., Frost, G., Skamarock, W. C., Eder, B. Fully coupled "online" chemistry within the WRF model. *Atmos. Environ*. **39**, 6957–6975 (2005).
6. Emmons, L. K. et al. Description and evaluation of the Model for Ozone and Related chemical Tracers, version 4 (MOZART-4). *Geosci. Model. Dev*. **3**, 43–67 (2010).
7. Guenther, A. B. et al. The Model of Emissions of Gases and Aerosols from Nature version 2.1 (MEGAN2.1): an extended and updated framework for modeling biogenic emissions. *Geosci. Model. Dev*. **5**, 1471–1492 (2012).
8. Wiedinmyer, C. et al. The Fire INventory from NCAR (FINN): a high resolution global model to estimate the emissions from open burning. *Geosci. Model. Dev*. 4, 625–641 (2011).
9. Zaveri, R. A., & Peters, L.K. A new lumped structure photochemical mechanism for large-scale applications. *J. Geophys. Res. Atmos*. **104**, 30387–30415 (1999).
10. Zaveri, R. A., Easter, R. C., Fast, J.D., & Peters, L. K. Model for simulating aerosol interactions and chemistry (MOSAIC). *J. Geophys. Res. Atmos*. **113**, 1395–1400 (2008).
11. Stockwell, W. R., Middleton, P., Chang, J. S., & Tang, X. The second generation regional acid deposition model chemical mechanism for regional air quality

modeling. *J. Geophys. Res. Atmos*. **95**, 16343–16367 (1990).

12. Schell, B., Ackermann, I. J., Hass, H., Binkowski, F. S., & Ebel, A. Modeling the formation of secondary organic aerosol within a comprehensive air quality model system. *J. Geophys. Res*. **106**, 28275–28293 (2001).
13. Yang, J. H., Kang, S. C., Ji, Z. M. Sensitivity analysis of chemical mechanisms in the WRF-chem model in reconstructing aerosol concentrations and optical properties in the Tibetan plateau. *Aerosol. Air. Qual. Res*. **18**, 505–521 (2018).
14. Gery, M. W., Whitten, G. Z., Killus, J. P. and Dodge, M .C. A photochemical kinetics mechanism for urban and regional scale computer modeling. *J. Geophys. Res*. **94**, 12925–12956 (1989).
15. Demore, W. B., Margitan, J. J., Molina, M. J., Watson, R. T., Golden, D. M., Hampson, R. F., Kurylo, M. J., Howard, C. J. & Ravishankara, A. R. Chemical kinetics and photochemical data for use in stratospheric modeling. National Aeronautics and Space Administration, Jet Propulsion Laboratory, Pasadena, Calif, pp. 1135–1151(1994).
16. Chen, S. H., & Sun, W. Y. A one-dimensional time dependent cloud model. *J. Meteor. Soc. Japan*. **80**, 99–118 (2002).
17. Kain, J. S. The Kain–Fritsch convective parameterization: An update. *J. Appl. Meteor*. **43**, 170–181 (2004).
18. Hong, S. Y., Yign, N., Jimy, D. A new vertical diffusion package with an explicit treatment of entrainment processes. *Mon. Wea. Rev*. **134**, 2318–2341 (2006).
19. Huffman, G. J., Adler, R. F., Arkin, P., … & Schneider, U. The Global Precipitation Climatology Project (GPCP) Combined Precipitation Dataset. *Bull. Amer. Meteor. Soc*. **78**, 5–20 (1997).
20. New, M., Lister, D., Hulme, M., & Makin, I. A high-resolution data set of surface climate over global land areas. *Clim. Res*. **21**, (2002).
21. Weedon, G. P., Balsamo, G., Bellouin, N., Gomes, S., Best, M. J., Viterbo, P. The WFDEI meteorological forcing data set: WATCH Forcing Data methodology applied to ERA-Interim reanalysis data. *Water. Resource. Res*. **50**, 7505–7514 (2014).
22. Ji, Z. M., Kang, S. C., Cong, Z. Y., Zhang, Q. G., & Yao, T. D. Simulation of carbonaceous aerosols over the Third Pole and adjacent regions: Distribution, transportation, deposition, and climatic effects. *Clim. Dynam*. **45**, 2831–2846

(2015).

23. Ji, Z., Kang, S., Zhang, D., Zhu, C., Wu, J. & Xu, Y. Simulation of the anthropogenic aerosols over south asia and their effects on indian summer monsoon. *Clim. Dynam*. **36**, 1633–1647 (2011).
24. Zheng, Y., Kiran, A., Jerold, A. et al. Improving High-Resolution Weather Forecasts Using the Weather Research and Forecasting (WRF) Model with an Updated Kain–Fritsch Scheme. *Mon. Weather. Rev*. **144**, 833–860 (2016).
25. Yang, J. H., Kang, S. C., Ji, Z. M., Chen, D. L. Modeling the origin of anthropogenic black carbon and its climatic effect over the Tibetan plateau and surrounding regions. *J. Geophys. Res.-Atmos*. **123**, 671–692 (2018).
26. Yang, J. H., Kang, S. C., Ji, Z. M. Critical contribution of south Asian residential emissions to atmospheric black carbon over the Tibetan plateau. *Sci. Total. Environ*. **709**, 135923 (2020).
27. Li, N., He, Q., Tie, X., Cao, J. J., Liu, S. X., Wang, Q. W., et al. Quantifying sources of elemental carbon over the Guanzhong Basin of China: a consistent network of measurements and WRF-Chem modeling. *Environ. Pollut*. **214**, 86–93 (2016a).
28. Li, Y. J., Zhang, L., Cao, X. J., Yun, Y., & Shi, J. S. Property of black carbon con centration over urban and suburban of Lanzhou. *China Environmental Science*, **34**, 1397–1403 (2014). (In Chinese)
29. Liu, X. C., Zhong, Y. T., He, Q., Yang, X. H., & Ali, M. Change character of black carbon concentration in atmosphere in Urumqi and the effect factors. *Desert and Oasis Meteorology*, **7**, 36–42 (2013) (in Chinese)
30. Li, C. L., Chen, P. F., Kang, S. C., Yan, F. P., Hu, Z. F., Qu, B., et al. Concentrations and light absorption characteristics of carbonaceous aerosol in $PM_{2.5}$ and PM10 of Lhasa city, the Tibetan Plateau. *Atmos. Environ*. **127**, 340–346 (2016b).
31. Chen, X. T., Kang, S. C., Cong, Z. Y., Yang, J. H., & Ma, Y. M. Concentration, temporal variation and sources of black carbon in the mount everest region retrieved by realtime observation and simulation. *Atmos. Chem. Phys*. **18**, 12859–12875 (2018).
32. Wan, X., Kang, S., Wang, Y., Xin, J., Liu, B., Guo, Y., et al. Size distribution of carbonaceous aerosols at a high-altitude site on the central Tibetan Plateau (Nam

Co Station, 4730ma.s.l.). *Atmos. Res*. **153**, 155–164 (2015).

33. Zhao, Z., Cao, J., Shen, Z., Huang, R. J., Hu, T., Wang, P., et al. Chemical composition of $PM_{2.5}$ at a high-altitude regional background site over Northeast of Tibet Plateau. *Atmos. Pollut. Res*. **5**, 815–823 (2015).
34. Zhao, S., Ming, J., Xiao, C., Sun, W., & Qin, X. A preliminary study on measurements of black carbon in the atmosphere of northwest Qilian Shan. *J. Environ. Sci*. **24**, 152–159 (2012).
35. Wang, M., Xu, B., Wang, N., Cao, J., Tie, X., et al. Two distinct patterns of seasonal variation of airborne black carbon over Tibetan Plateau. *Sci. Total. Environ*. **573**, 1041–1052 (2016).
36. Cao, J. J., Xu, B. Q., He, J. Q., Liu, X. Q., Han, Y. M., Wang, G. H., et al. Concentrations, seasonal variations, and transport of carbonaceous aerosols at a remote Mountainous region in western China. *Atmos. Environ*. **43**, 4444–4452 (2009).
37. Marinoni, A., Cristofanelli, P., Laj, P., Duchi, R., Calzolari, F., Decesari, S., et al. Aerosol mass and black carbon concentrations, a two year record at NCO-P (5079 m, Southern Himalayas). *Atmos. Chem. Phys*. **10**, 8551–8562 (2010).
38. Ram, K., Sarin, M. M., & Hegde, P. Long-term record of aerosol optical properties and chemical composition from a high-altitude site (Manora Peak) in Central Himalaya. *Atmos. Chem. Phys*. **10**, 11791–11803 (2010).
39. Begum, B. A., Hossain, A., Nahar, N., Markwitz, A., & Hopke, P. K. Organic and black carbon in $PM_{2.5}$ at an urban site at Dhaka, Bangladesh. *Aerosol. Air. Qual. Res*. **12**, 1062–1072 (2012).
40. Safai, P. D., Raju, M. P., Budhavant, K. B., Rao, P. S. P., & Devara, P. C. S. Long term studies on characteristics of black carbon aerosols over a tropical urban station Pune, India. *Atmos. Res*. **132–133**, 173–184 (2013).
41. Zhang, M., Zhao, C., Cong, Z., Du, Q., Xu, M., Chen, Y., Chen, M., Li, R., Fu, Y., Zhong, L., Kang, S., Zhao, D., Yang, Y. Impact of topography on black carbon transport to the southern Tibetan Plateau during the pre-monsoon season and its climatic implication, Atmos. Chem. Phys., **20**, 5923–5943 (2020).
42. Kang, S., Zhang, Y., Chen, P., Guo, J., Zhang, Q., Cong, Z., ... & Li, C. Black carbon and organic carbon dataset over the Third Pole. *Earth Syst. Sci. Data*. **14**, 683–707 (2021).

43. Ji, Z. M. Modeling black carbon and its potential radiative effects over the Tibetan Plateau. *Adv. Clim. Change. Res*. **7**, 139–144 (2016).

44. Joshi, H., Naja, M., Singh, K., Kumar, R., Bhardwaj, P., Babu, S. S., Satheesh, S., Moorthy, K. K., Chandola, H. Investigations of aerosol black carbon from a semi-urban site in the Indo-Gangetic Plain region. *Atmos. Environ*. **125**, 346–359 (2016).

45. Nair, V. S., Solmon, F., Giorgi, F., Mariotti, L., Babu, S. S., Moorthy, K. K. Simulation of South Asian aerosols for regional climate studies. *J. Geophys. Res.-Atmos.* **117**, (2012).

46. Alvarado, M. J., Winijkul, E., Adams-Selin, R., Hunt, E., ... & Mosier, T. M. Sources of black carbon deposition to the Himalayan glaciers in current and future climates. *J. Geophys. Res.-Atmos.* **123**: 7482–7505 (2018).

47. Kumar, R., Barth, M. C., Nair, V. S., Pfister, G. G., Suresh Babu, S., Satheesh, S. K., Krishna Moorthy, K., Carmichael, G. R., Lu, Z., Streets, D. G. Sources of black carbon aerosols in South Asia and surrounding regions during the Integrated Campaign for Aerosols, Gases and Radiation Budget (ICARB). *Atmos. Chem. Phys*. **15**, 5415–5428 (2015).

48. Pan, X., Chin, M., Gautam, R., Bian, H., Kim, D., Colarco, P. R., Diehl, T. L., Takemura, T., Pozzoli, L., Tsigaridis, K. A multi-model evaluation of aerosols over South Asia: common problems and possible causes. *Atmos. Chem. Phys*. **15.10**, 5903–5928 (2015).

49. Lee, S. Y., & Wang, C. The response of the South Asian summer monsoon to temporal and spatial variations in absorbing aerosol radiative forcing. *J. Clim*. **28**, 6626-6646 (2015).

50. Ramanathan, V. et al. Atmospheric brown clouds: impacts on south asian climate and hydrological cycle. *P. Natl. Acad. Sci. U.S.A*. 1**02**, 5326–5333 (2005).

51. Chung, C. E., Ramanathan, V., & Kiehl, J. T. Effects of the South Asian absorbing haze on the northeast monsoon and surface–air heat exchange. *J. Clim*. **15**, 2462–2476 (2002).

52. Lau, K. M., Kim, M. K., Kim. K. M. Asian monsoon anomalies induced by aerosol direct effects. *Clim. Dyn*. **26**, 855–864 (2006).

53. Lau, K. M. & Kim, K. M. Observational relationships between aerosol and Asian monsoon rainfall, and circulation. *Geophys. Res. Lett*. **33**, L21810 (2006).

54. Soni, P., Tripathi, S. N. & Srivastava, R. Radiative effects of black carbon aerosols on Indian monsoon: a study using WRF-Chem model. *Theor. Appl. Climatol*. **132**, 115–134 (2018).
55. Dong, W. et al. Summer rainfall over the southwestern Tibetan Plateau controlled by deep convection over the Indian subcontinent. *Nat. Commun*. **7**, 10925 (2016).
56. Radić, V. & Hock, R. Regionally differentiated contribution of mountain glaciers and ice caps to future sea-level rise. *Nat. Geosci*. **4**, 91–94 (2011).
57. Radić, V., Bliss, A., Beedlow, A. C., Hock, R., Miles, E., Cogley, J. G. Regional and global projections of twenty-first century glacier mass changes in response to climate scenarios from global climate models. *Clim. Dyn*. **42**, 37–58 (2014).
58. Kraaijenbrink, P. D. A., Bierkens, M. F. P., Lutz, A. F. & Immerzeel, W. W. Impact of a global temperature rise of 1.5 degrees Celsius on Asia's glaciers. *Nature* **549**, 257–260 (2017).
59. Wang, Z., Duan, A., Yang, S. & Ullah, K. Atmospheric moisture budget and its regulation on the variability of summer precipitation over the Tibetan Plateau. *J. Geophys. Res. Atmos*. **122**, 614–630 (2017).
60. Lin, H. B., You, Q. L., Jiao, Y., Ming, J. Z. Water vapor transportation and its influences on precipitation in summer over Qinghai-Xizang Plateau and its surroundings. *Plateau Meteorology*, **35**, 309–317 (2016) (in Chinese).
61. Curio, J., Maussion, F. & Scherer, D. A 12-year high-resolution climatology of atmospheric water transport over the Tibetan Plateau. *Earth. Syst. Dyn*. **6**, 109–124 (2015).
62. Ramanathan, V. & Carmichael, G. Global and regional climate changes due to black carbon. *Nat. Geosci*. **1**, 221–227(2008).
63. Menon, S., Hansen, J., Nazarenko, L. & Luo, Y. Climate effects of black carbon aerosols in china and india. *Science* **297**, (2002).
64. Fan, J. et al. Microphysical effects determine macrophysical response for aerosol impacts on deep convective clouds. *Proc. Natl. Acad. Sci. U.S.A*. **110**, E4581–E4590 (2013).
65. Rosenfeld, D. et al. Flood or drought: How do aerosols affect precipitation?, *Science*. **321**, 1309–1313 (2008).
66. Yuan, T., Li, Z., Zhang, R. & Fan, J. Increase of cloud droplet size with aerosol optical depth: An observation and modeling study. *J. Geophys. Res. Atmos*. **113**,

D04201 (2008).

67. Yao, T. et al. Different glacier status with atmospheric circulations in Tibetan Plateau and surroundings. *Nat. Clim. Change* **2**, 663–667 (2012).

68. Brun, F., Berthier, E., Wagnon, P., et al. A spatially resolved estimate of High Mountain Asia glacier mass balances from 2000 to 2016. *Nat. Geosci*. **10**, 668-673, (2017).

69. Kääb, A., Berthier, E., Nuth, C., Gardelle, J. & Arnaud, Y. Contrasting patterns of early twenty-first-century glacier mass change in the Himalayas. *Nature* **488**, 495–498 (2012).

70. Wang, R., Liu, S., Shangguan, D., Radić, V. & Zhang, Y. Spatial heterogeneity in glacier mass-balance sensitivity across High Mountain Asia. *Water*. **11**, 776 (2019).

71. Gardner, A. S., Moholdt, G., Cogley, J. G., Wouters, B., Arendt, A. A., Wahr, J., Berthier, E., Hock, R., Pfeffer, W. T., Kaser, G. A reconciled estimate of glacier contributions to sea level rise: 2003 to 2009. *Science* **340**, 852–857 (2013).

72. Jacob, T., Wahr, J., Pfeffer, W. T., Swenson, S. Recent contributions of glaciers and ice caps to sea level rise. *Nature* **482**, 514–518 (2012).

73. Immerzeel, W.W., Beek, L. P. H. & Bierkens, M. F. P. Climate change will affect the Asian water towers. *Science* **328**, 1382–1385 (2010).

74. Bolch, T. et al. The state and fate of Himalayan glaciers. *Science* **336**, 310–314 (2012).

75. Feng, L. & Zhou, T. Water vapor transport for summer precipitation over the Tibetan Plateau: Multidata set analysis. *J. Geophys. Res. Atmos*. **117**, 20114 (2012).

76. Turner, A. G. & Annamalai, H. Climate change and the South Asian summer monsoon. *Nat. Clim. Change.* **2**, 587–595 (2012).
